# Cloning, Characteristics, and Functional Analysis of Rabbit NADPH Oxidase 5

**DOI:** 10.3389/fphys.2016.00284

**Published:** 2016-07-19

**Authors:** Feng Chen, Caiyong Yin, Christiana Dimitropoulou, David J. R. Fulton

**Affiliations:** ^1^Department of Forensic Medicine, Nanjing Medical UniversityNanjing, Jiangsu, China; ^2^Vascular Biology Center, Medical College of Georgia at Augusta UniversityAugusta, GA, USA; ^3^Frank Reidy Research Center for Bioelectrics, Old Dominion UniversityNorfolk, VA, USA

**Keywords:** Nox5, rabbit, NADPH oxidase, superoxide, phylogenetic analysis

## Abstract

**Background:** Nox5 was the last member of the Nox enzyme family to be identified. Functionally distinct from the other Nox isoforms, our understanding of its physiological significance has been hampered by the absence of Nox5 in mouse and rat genomes. Nox5 is present in the genomes of other species such as the rabbit that have broad utility as models of cardiovascular disease. However, the mRNA sequence, characteristics, and functional analysis of rabbit Nox5 has not been fully defined and were the goals of the current study.

**Methods:** Rabbit Nox5 was amplified from rabbit tissue, cloned, and sequenced. COS-7 cells were employed for expression and functional analysis via Western blotting and measurements of superoxide. We designed and synthesized miRNAs selectively targeting rabbit Nox5. The nucleotide and amino acid sequences of rabbit Nox5 were aligned with those of putative rabbit isoforms (X1, X2, X3, and X4). A phylogenetic tree was generated based on the mRNA sequence for Nox5 from rabbit and other species.

**Results:** Sequence alignment revealed that the identified rabbit Nox5 was highly conserved with the predicted sequence of rabbit Nox5. Cell based experiments reveal that rabbit Nox5 was robustly expressed and produced superoxide at rest and in a calcium and PMA-dependent manner that was susceptible to superoxide dismutase and the flavoprotein inhibitor, DPI. miRNA-1 was shown to be most effective in down-regulating the expression of rabbit Nox5. Phylogenetic analysis revealed a close relationship between rabbit and armadillo Nox5. Rabbit Nox5 was relatively closely related to human Nox5, but lies in a distinct cluster.

**Conclusion:** Our study establishes the suitability of the rabbit as a model organism to further our understanding of the role of Nox5 in cardiovascular and other diseases and provides new information on the genetic relationship of Nox5 genes in different species.

## Introduction

The NADPH family of oxidases, also referred to as the Noxes, are transmembrane oxidoreductases that utilize NADPH to transport electrons across biological membranes to reduce oxygen to produce superoxide and other types of reactive oxygen species (ROS; Bedard and Krause, 2007; Kawahara and Lambeth, 2008). There are seven related isoforms including Nox1, Nox2, Nox3, Nox4, Nox5, Duox1, and Duox2 which share the same basic organizational layout, but produce different types and amounts of ROS via different mechanisms in different tissues to enact different functions. Nox enzmyes have important physiological roles in the immune system, transduction of cellular signaling and the formation of otoconia. The overexpression and inappropriate regulation of Nox enzymes has also been observed to contribute to pathophysiologic processes. In the cardiovascular, renal, and pulmonary systems, increased expression and activity of Nox1, Nox2, and Nox4 have been shown underlie disease processes such as cellular proliferation and tumorigenesis (Bedard and Krause, 2007; Lambeth et al., 2007). In the vestibular system, Nox3 plays an important role in the gravity sensing function of inner ear (Paffenholz et al., 2004; Nakano et al., 2008).

In 2001, two independent research groups discovered a novel protein with significant homology to Nox1 and Nox2 (Bánfi et al., 2001; Cheng et al., 2001). This protein was named Nox5 as it was discovered after Nox4. Various human tissues express Nox5, including testis, spleen, cornea, and vascular tissue (Fulton, 2009; O'Brien et al., 2009). Nox5 has five splice variants: Nox5 α, β, δ, γ, and ε or Nox5-S, a truncated variant. In humans, the gene for Nox5 is located on chromosome 15. The C-terminus of Nox5 contains binding regions for FAD and NADPH which are highly conserved with the other Nox isoforms (Paffenholz et al., 2004; Kawahara and Lambeth, 2008). Except for Nox5-S, all of the splice variants of Nox5 possess N-terminal EF hands which are calcium binding domains (BelAiba et al., 2007; Fulton, 2009; Pandey et al., 2012). In contrast to the regulation of Nox1-4 which occurs through interaction with other proteins including p22^phox^, p47^phox^, p67^phox^, p40^phox^, and Rac (Ambasta et al., 2006; Hordijk, 2006; Ushio-Fukai, 2006; Bedard and Krause, 2007; Lambeth et al., 2007; Karimi et al., 2014), calcium is essential for activating and regulating Nox5 activity and this occurs via the N-terminal calcium-binding EF hands (Bánfi et al., 2001, 2004; Cheng et al., 2001; Tirone et al., 2010). Rac1 was reported to be important in the activation of Nox5-S in esophageal adenocarcinoma (FLO EA) cells but this remains controversial (Montezano et al., 2010; Hong et al., 2011). We and others have recently discovered a number of novel regulatory mechanisms that influence the enzyme activity of Nox5, including phosphorylation (Pandey and Fulton, 2011; Pandey et al., 2011a; Chen et al., 2014a), S-nitrosylation (Qian et al., 2012), and SUMOylation (Pandey et al., 2011b), protein:protein interactions (Chen et al., 2014b) and molecular chaperones (Chen et al., 2011, 2012, 2015a).

Genes encoding homologs of Nox5 are found in both invertebrates and vertebrates. Despite being an archetypal Nox enzyme, Nox5 was lost from the genomes of rodents (Kawahara et al., 2007). The reason for this, if any, and the physiological and pathophysiological significance of Nox5 awaits further clarification. In humans, high expression of Nox5 which is accompanied by excess ROS has been reported in both cancer (Brar et al., 2003; Kamiguti et al., 2005; Fu et al., 2006; Si et al., 2008) and cardiovascular disease (Guzik et al., 2008). We previously reported that several single nucleotide polymorphisms (SNPs) within the coding region of Nox5 result in truncated or abolished Nox5 activity in humans. Some of these SNPs occur with high frequency in certain human populations (Wang et al., 2014), but relationships between the loss of Nox5 function and human health have not yet been identified. In addition to rodents, rabbits have been widely used as models of human cardiovascular disease. Recently, a research group has shown that Nox5 is expressed in lagomorphs using human primers for Nox5 to amplify a partial mRNA sequence which was confirmed to be specific to Nox5 in rabbit corneal stromal cells (Rizvi et al., 2012). However, the full length of rabbit Nox5 mRNA sequences and the conservation or the phylogenetic relationship of human Nox5, rabbit Nox5 and other Nox5 mRNA sequences remain to be determined, furthermore whether the gene for Nox5 is functional and the regulatory mechanism of Nox5 in rabbits or whether antibodies specific for human Nox5 can recognize the rabbit isoform are not yet known.

In this study, we cloned and characterized the rabbit Nox5, confirming that it is functional, responds to both calcium-dependent, and phosphorylation-dependent signals and can be detected by polyclonal antibodies that recognize the human Nox5. In addition, we aligned the rabbit Nox5 sequence with the Nox5 sequence from 19 other mammalian or non-mammalian species and constructed a phylogenetic tree. This study furthers our understanding of the origin of Nox5 and more significantly validates the utility of the rabbit as a model system to study the importance of Nox5 under physiology and pathophysiology conditions.

## Materials and methods

### Animals

Rabbit testis tissues were obtained from experimental discards and no live animals were used for this study. All experiments were conducted in accord with the National Institutes of Health (NIH) Guide for the Care and Use of Laboratory Animals and approved and monitored by the Augusta University Institutional Animal Care and Use Committee (Augusta, GA).

### Cell cultures and transfection

As described previously (Chen et al., 2011, 2012), COS-7 cells were grown in Dulbecco's modified Eagle's medium (DMEM) containing streptomycin (100 mg/ml), penicillin (100 U/ml), and 10% (v/v) fetal bovine serum. Lipofectamine 3000 reagent was used to transfect COS-7 cells according to the manufacturer's instructions (Invitrogen, Carlsbad, CA). In brief, COS-7 cells were grown in a 12-well plate and at ~90% confluency were transfected using a plasmid DNA-lipid mixture of 1 μg plasmid/well at the recommended ratio of 1 μg DNA: 2 μL lipofectamine. Gene expression was analyzed 48 h post-transfection.

### Nox5 cloning, sequencing, and sequence analysis

Total RNA was extracted from the testis of rabbit, and reverse transcribed to cDNA using Bio-Rad RT-PCR kit. PCR was performed in a 25 μL reaction volume using Bio-Rad IQ5 thermometer. Primers were initially selected based on homology to human and then extended using primer walking to obtain a full length coding sequence which was cloned into the pcDNA3.1 directional topo expression vector kit. Partial and full length clones were sent to Genewiz (South Plainfield, NJ) for sequencing and the resulting sequences aligned. ExPASy (http://ca.expasy.org/tools/dna.html), Prosite (http://prosite.expasy.org), and CDD algorithms (http://www.ncbi.nlm.nih.gov/Structure/cdd/cdd.shtml) were used to translate as well as to detect the presence of functional motifs and conserved regions in the protein. Predicted rabbit Nox5, human Nox5 as well as Nox5 sequences from other species were downloaded from the National Center for Biotechnology Information (NCBI) (http://ncbi.nlm.nih.gov) and Genome Browser (http://genome.ucsc.edu) from University of California Santa Cruz (UCSC). Bioedit software was utilized for DNA and protein sequences alignment and Mega 4 software ver. 4.0 was used for illustrating dendrogram by the neighbor-joining method (Tamura et al., 2007).

### Adenoviral generation and transduction

Control (RFP), rabbit HA-Nox5 and rabbit Flag-Nox5 adenoviruses were generated as described (Qian et al., 2012). MicroRNA targeting different regions of the Nox5 mRNA were made by using BLOCK-iT™ Pol II miR RNAi Expression Vector Kit according to the manufacture's instructions. COS-7 cells were seeded in 12-well plates, and the next day cells were transduced at a multiplicity of infection (MOI) of 30 in total.

### Western blotting

Phosphate-buffered saline (PBS) was used to wash cells twice followed by lysis in Laemmli sample buffer containing 50 mM Tris–HCl, pH 6.8, 30% glycerol, 2% SDS, 6% β-mercaptoethanol, and 0.02% bromphenol blue at room temperature. Cell lysates were denatured at 100°C for 5 min, clarified at 14,000xg for 10 min at 4°C and then size-fractionated using 5–20% SDS-polyacrylamide gel electrophoresis. Separated proteins were transferred to nitrocellulose membranes and immunoblotted with the following antibodies at the final concentration of 1 ug/ml: anti-Nox5 (Jagnandan et al., 2007), anti-HA (Roche), anti-Flag (Cell Signaling Technology), and GAPDH (Santa Cruz Biotechnology). The Nox5 polyclonal antibody has been described previously (Jagnandan et al., 2007) and recognizes the sequence APRPRPRRPRQLTRA (aa170–184 on human Nox5β).

### Measurement of superoxide production

Transfected cells were seeded into 96-well white tissue culture plates (Thermo Fisher Scientific) at a density of around 5 × 10^4^. Prior to the addition of agonists, cells were incubated at 37°C in phenol-free Dulbecco's modified Eagle's medium (Sigma-Aldrich, St. Louis, MO) together with the luminol analog, L-012 (Wako Pure Chemicals, Tokyo, Japan, 400 μM) for 20 min. Luminescence was quantified using a Lumistar Galaxy (BMG Labtech, Durham, NC) luminometer and the signal recorded in the form of relative light units (RLU). The specificity of L-012 for Nox-derived superoxide was confirmed by (i) transfecting COS-7 cells with a control plasmid, RFP, that does not produce superoxide (ii) co-incubation with diphenylene iodonium (DPI) a NADPH oxidase inhibitor or (iii) superoxide dismutase (SOD) which specifically scavenges superoxide (Chen and Fulton, 2010; Chen et al., 2011, 2012, 2014a, 2015a,b). The levels of luminescence in RFP transfected cells were undetectable.

### Statistical analysis

Data were displayed as means ± SE and statistical differences determined using Instat software (GraphPad Software Inc., San Diego, CA) via either a two-tailed student's *t*-test or ANOVA with a *post-hoc* test. Significant difference was set as *p* < 0.05.

## Results

### Sequence of rabbit Nox5

**Figure 1:**
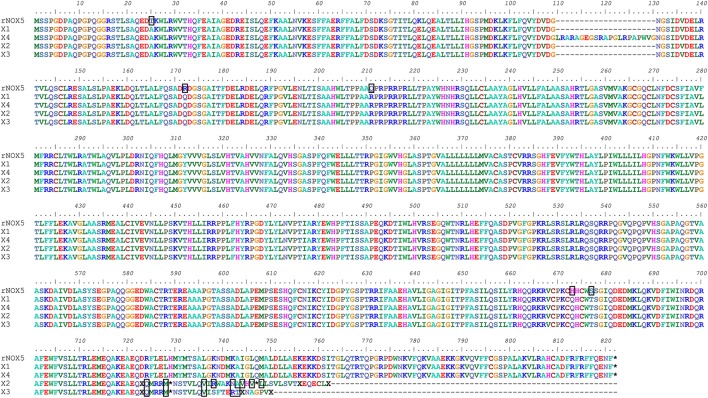
**Alignment of cloned rabbit Nox5 with the predicted sequences for rabbit Nox5 isoforms (X1, X2, X3, and X4) based on genomic sequencing**. The amino acids outlined by a black box represent heterogeneity between the cloned rabbit Nox5 and the predicted isoforms. The predicted sequences of rabbit Nox5 isoforms X1 (XM_008249804.1), X2 (XM_008249805.1), X3 (XM_008249806.1), and X4 (XM_008249807.1) were obtained from NCBI (Gene ID: 100301540), and aligned with the sequence of cloned Nox5.

The mRNA and protein sequences of rabbit (oryctolagus cuniculus) Nox5 were analyzed using Bioedit software (Supplemental Figure 1). Variants of single nucleotide and amino acid are shown. Comparison of the cloned Nox5 with the predicted Nox5 rabbit isoforms in NCBI reveals that most of the coding region of the cloned rabbit Nox5 is consistent with predicted sequences. In addition, an alignment of the amino acid sequence of the cloned Nox5 and predicted Nox5 oryctolagus cuniculus isoforms X1, X2, X3, and X4 was conducted to visually display mismatched amino acids (marked with a black box, Figure 1). The alignment detected a difference of 21 adjacent amino acids at location aa110–130 of the X4 isoform vs. the cloned rabbit Nox5. In addition, 5 mismatched amino acids were detected in the cloned rabbit Nox5 vs. the other 4 predicted Nox5 isoforms. These 5 amino acids were T25 (Threonine) of sequenced rabbit Nox5 vs. A (Alanine) at the 25th position, R151 (Arginine) vs. Q (Glutamine) at the 172st position, Q190 (Glutamine) vs. R (Arginine) at the 211st position, H652 (Histidine) vs. Q (Glutamine) at the 673rd position, and S656 (Serine) vs. T (Threonine) at the 677th position, respectively. Furthermore, at the 724th position toward the end of C-flanking sequences, the predicted rabbit Nox5 isoforms X2 and X3 displayed a greater difference compared to the cloned rabbit Nox5. Data also indicate that the predicted X1 isoform had the closest homology with the cloned rabbit Nox5. In comparison to the human Nox5 isoforms, cloned rabbit Nox5 was most similar to human Nox5 alpha (V1) in that it possesses an N-terminal extension that is not present in Nox5 beta (V2) and the overall identity of rabbit Nox5 vs. the human isoforms was ~85% (Supplemental Figure 2).

### Validation of the expression and function of rabbit Nox5 in mammalian cells

**Figure 2:**
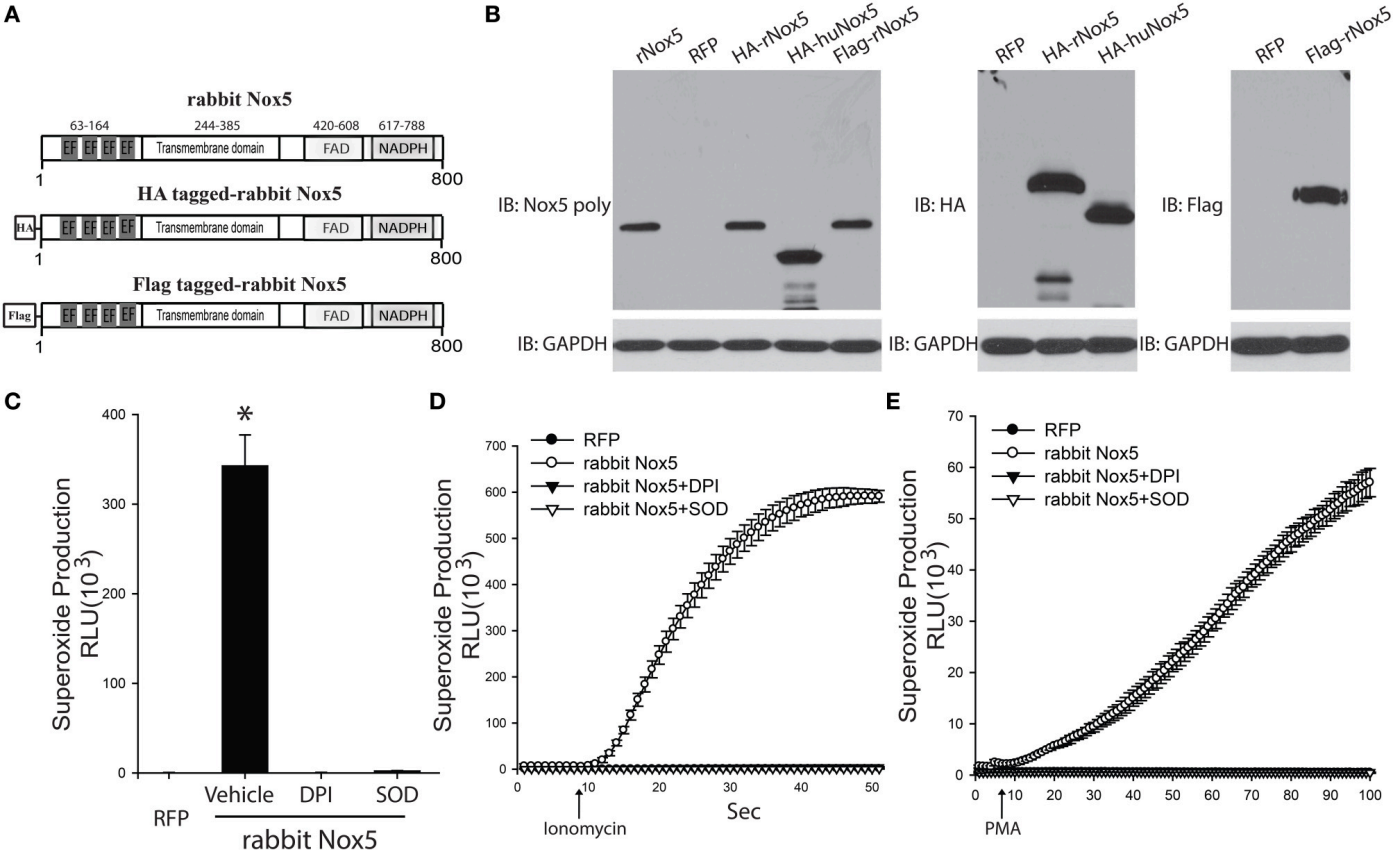
**Expression and function of cloned rabbit Nox5 gene. (A)** Schematic representation of rabbit Nox5, rabbit HA-Nox5, and Flag-Nox5 constructs. **(B)** COS-7 cells were transfected with plasmids encoding rabbit Nox5, rabbit HA-Nox5, Flag-Nox5, human Nox5beta and a control gene (RFP), and cell lysates immunoblotted for Nox5, HA, Flag and GAPDH. Results are representative of at least 3 separate experiments. **(C–E)** COS-7 cells transfected with cDNAs encoding RFP or rabbit Nox5 and incubated with or without DPI (10 μM) and SOD (100 U/ml) for 30 min. Superoxide was measured using L-012 chemiluminescence under basal, ionomycin, and PMA stimulated conditions. ^*^different from RFP, *p* < 0.05 (*n* = 5–6).

In order to determine whether the cloned rabbit Nox5 expresses a functional protein, we transfected COS-7 cells with the rabbit Nox5 plasmid and measured its expression and activity by Western blotting and superoxide-specific chemiluminescence. Conserved regions as well as characteristic motifs of the Nox5 gene are shown in Figure 2A. Rabbit Nox5 was also epitope tagged with both a HA or Flag tag on the N-terminus to facilitate detection of expression by Western blotting. We found that rabbit Nox5 protein was strongly expressed in COS-7 cells at 48 h after transfection. In addition, the molecular weight of WT rabbit Nox5 protein was the same as the epitope tagged HA-rabbit Nox5 and Flag-rabbit Nox5 and there was no band from cells transfected with the control plasmid RFP (Figure 2B). However, the molecular weight of rabbit Nox5 was higher than that of human Nox5beta and this result is consistent with the predicted higher molecular weight of rabbit Nox5 (rabbit: 800aa, ~90 kDa, human Nox5beta: 719aa, ~82 kDa). Next, to determine whether the expressed rabbit Nox5 protein is functional, we measured superoxide production in transfected cells. COS-7 cells were transfected with RFP or rabbit Nox5 and then treated with vehicle, DPI or SOD and superoxide was measured using L-012 enhanced chemiluminescence. As shown in Figures 2C–E, the expressed rabbit Nox5 produced large quantities of superoxide that was effectively inhibited by DPI or SOD under both resting conditions (basal) and following stimulation with ionomycin (a calcium-dependent stimulus) and PMA (a calcium-independent stimulus).

### Silencing of rabbit Nox5

**Figure 3:**
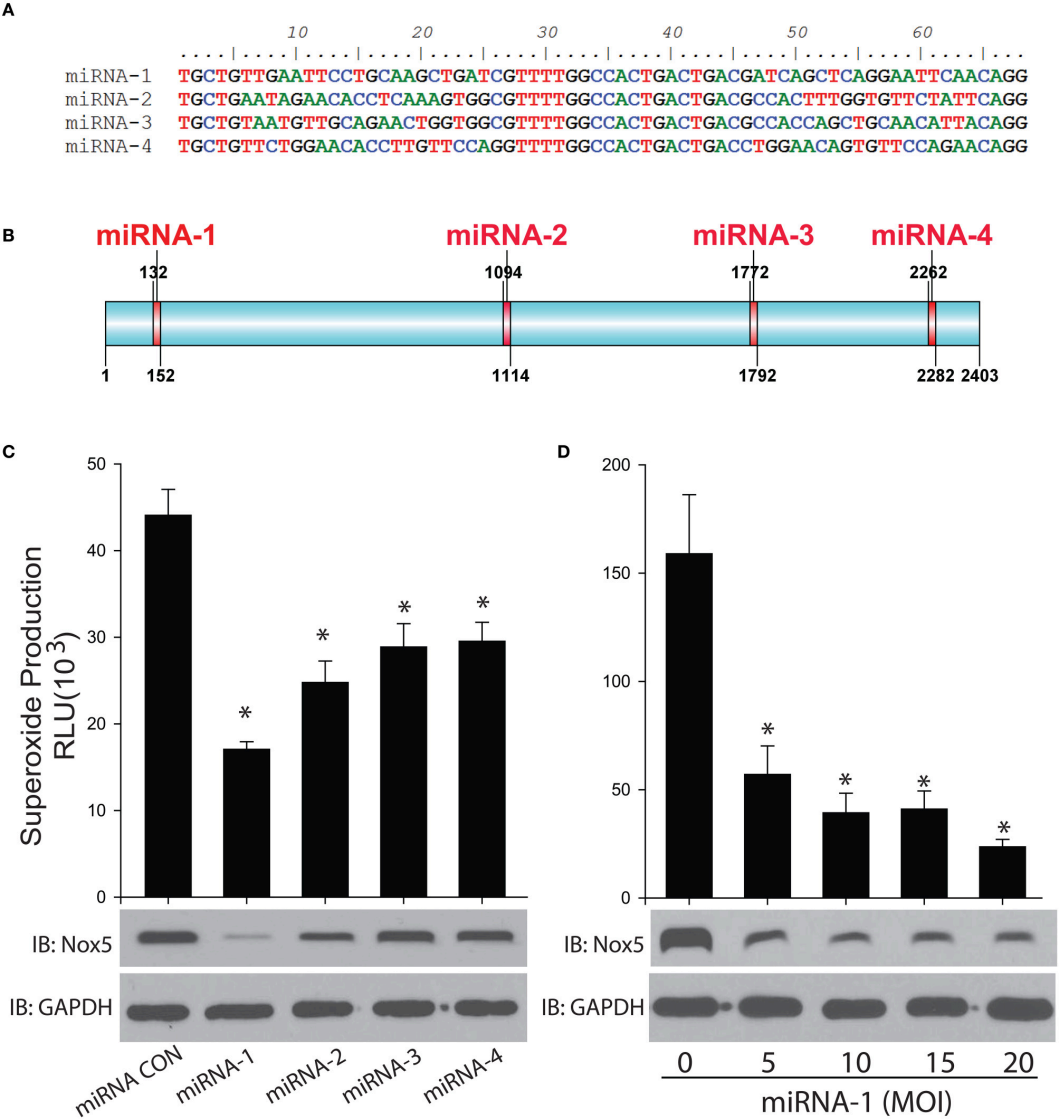
**Gene silencing of rabbit Nox5 by designed 4 miRNAs was determined by western blotting and superoxide measurement. (A,B)** Four miRNAs were designed using RNAi Designer and the resultant sequences and relative locations are shown in **(B)**. **(C)** Four miRNAs or **(D)** increasing amount of miRNA-1 were co-transduced with the rabbit Nox5 cDNA clone in cells (the total amount of miRNA used was balanced with control miRNA), the efficiency of gene silencing was determined by Western blotting and measurement of superoxide. ^*^different from control, *p* < 0.05 (*n* = 5–6).

Four miRNAs were designed to specifically bind to rabbit Nox5 mRNA to silence its expression in cells (Figures 3A,B). We found all 4 miRNAs decreased superoxide production as well as the protein expression of rabbit Nox5 with miRNA-1 exhibiting the best efficiency (Figure 3C). We then tested the dose effect relationship of miRNA-1, and found that it could dose-dependently decrease rabbit Nox5 protein expression (Figure 3D). These data suggest that rabbit Nox5 expression can be readily silenced by miRNAs and that targeting different regions may influence efficacy.

### Phylogenetic structure and evolutionary significance of rabbit Nox5

**Figure 4:**
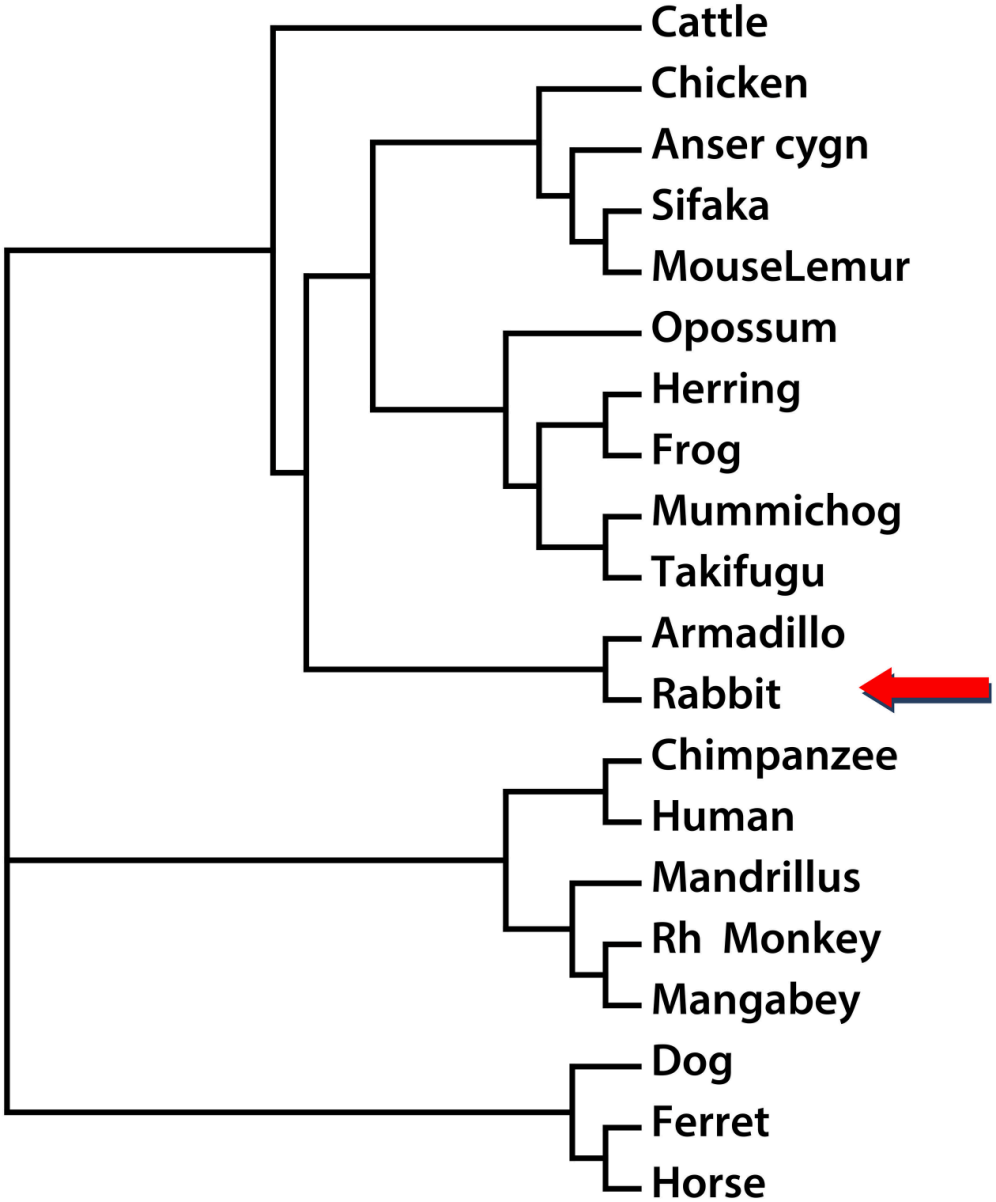
**Phylogenetic reconstruction using Nox5 sequences from different species**. The rabbit Nox5 sequence and sequences of Nox5 from 19 other species obtained from NCBI or UCSC were aligned, and a phylogenetic tree was drawn using the neighbor-joining method.

In order to establish the phylogenetic relationship between Nox5 in different species, the Neighbor-Joining method was utilized to create a phylogenetic tree based on the mRNA sequences of Nox5 in rabbit, Cattle, Chicken, Anser cygnoides (Swan goose), Sifaka, Mouse Lemur, Opossum, Herring, Frog, Mummichog, Takifugu, Armadillo, Chimpanzee, Human, Mandrillus, Rh Monkey, Mangabey, Dog, Ferret, and Horse (based on sequences obtained from Genbank. The accession numbers are listed in Supplemental Figure 3. Using Nox5 as a genetic marker, three main clusters are shown in the phylogenetic tree (Figure 4). The genus rabbit clustered with armadillo, which indicates a close genetic relationship between the rabbit and armadillo. In contrast human Nox5 has closer relationships with chimpanzee, mandrillus, Rh monkey, and mangabey and this data is consistent with the evolutionary pathway of respective species. The phylogenetic tree further suggests that monkeys would be the most closely related and therefore most appropriate animal model to study the role of Nox5-derived superoxide in cardiovascular disease, whereas rabbit and perhaps other models could be used when monkeys are impractical.

## Discussion

In the current study we report on the cloning and sequencing of rabbit Nox5. The major contributions to our knowledge are the mRNA sequence of rabbit Nox5 and its encoded protein, demonstration that rabbit Nox5 expresses well and functions similarly to human Nox5, identification of a polyclonal antibody that can recognize rabbit Nox5, identification of an optimal miRNA that can selectively silence rabbit Nox5 as well as establishing a phylogenetic relationship between rabbit Nox5 and that of other species. Given the absence of Nox5 from the genomes of rats and mice, the importance of Nox5 to the development of cardiovascular diseases, cancer and other diseases is, by comparison, poorly understood. Our findings provide a framework for future studies of Nox5 using rabbit models of human diseases.

The activity of human Nox5 primarily depends on the regulation by calcium binding to four N-terminal EF-hand modules (Bánfi et al., 2001, 2004; Jagnandan et al., 2007; Wei et al., 2010) and also the cooperation of various signal mechanisms including phosphorylation and protein: protein interactions involving chaperones such as Hsp90, caldmodulin and caveolin-1 (Tirone and Cox, 2007; Pandey et al., 2011a; Qian et al., 2012). The deduced amino acid sequence of rabbit Nox5 as well as the predicted rabbit Nox5 sequences reveals a high degree of conservation with numerous features previously identified in human Nox5. Rabbit Nox5 is a 6 transmembrane spanning protein that possesses C-terminal NADPH and FAD binding sites as well as 4 heme binding histidine residues and 4 EF hand motifs in the N-terminus. Consistent with human Nox5, expressed rabbit Nox5 produces abundant levels of superoxide as demonstrated by the ability of DPI and SOD to suppress L-012-dependent chemiluminescence and the almost complete absence of signal in control transfected cells. In humans, 5 isoforms of Nox5 have been identified (designated V1-V5 or α-ε) but only 2 of thee isoforms appear to be functional [V1(α), V2(β)]. The cloned rabbit Nox5 is most similar to the functional human Nox5α (V1). Whether the rabbit equivalents of the other Nox5 isoforms (V2–V5, β-ε) are expressed in rabbit cells remains to be determined.

In human Nox5, the binding of calcium to N-terminal EF hands evokes a conformational change that allows binding of the N-terminus to a putative C-terminal Regulatory EF-hand Binding Domain (REFBD, aa 638–661 Nox5 V2) which enables electron flow from the C to N-terminus for superoxide generation (Tirone et al., 2010). The equivalent region in rabbit Nox5 varies significantly from the human sequence at the amino acid level but the majority of the differences are conservative substitutions. In comparison to human Nox5, rabbit Nox5 contains a substitution at T512 (equivalent to T494 on human Nox5 beta) which is a previously identified phosphorylation site for PKC (Jagnandan et al., 2007). However, it is unlikely that this modification alone substantially affects the activity of rabbit Nox5 and this hypothesis is supported by the ability of PMA, which stimulates Nox5 activity by phosphorylation of T494 and other nearby residues, to robustly increase superoxide levels in cells expressing rabbit Nox5. This is also consistent with data showing that the selective mutation of single phosphorylation sites in the equivalent region of human Nox5 did not affect PMA-dependent increases in superoxide and that mutation of multiple phosphorylation sites is necessary to reduce activity (Jagnandan et al., 2007). The two other phosphorylation sites that lie in close proximity are S508 (equivalent to S490 hNox5beta) and S516 (S498 hNox5beta) which are conserved in rabbit Nox5. The cloned rabbit Nox5 also contains an insert right after the major PKC phosphorylation sites (PQPVHSGAPAQGTVAASKDAIVDLASYSEGPAQQGGEDWACTRTEREAAAPGTASSADLAPE) that is also present in the predicted sequences of rabbit Nox5. This region is absent in human Nox5 but present, albeit with less homology, in the sequences of ferret, whale and donkey Nox5. The function of this region, if any, is not yet known. However, it does not seem to interfere with enzyme activity and the ability of rabbit Nox5 to produce superoxide at rest or following calcium or phosphorylation-dependent stimuli (Rizvi et al., 2012). We and others have shown that Hsp90 (Heat shock protein 90) and co-chaperones, such as Hsp70, Hsp40, p23, and HOP can influence Nox5 stability and function by binding to a C-terminal region. In the presence of an Hsp90 inhibitor, the binding of Hsp90 and p23 is decreased while binding of Hsp70, Hop, and Hsp40 to Nox5 is increased. Functionally, Nox5 derived ROS production is reduced in cells where the expression of HOP, Hsp40, or p23 is reduced using siRNA or in cells with increased expression of Hsp70 (Chen et al., 2015a). Interestingly, the insertion identified in rabbit Nox5 is predicted to lie within the Hsp90 binding region (aa490–550, Nox5V2) but whether this impacts the binding of Hsp90 or other chaperones to rabbit Nox5 is not known. In addition, calmodulin and caveolin-1 have been shown to be allosteric regulators of Nox5 activity that, respectively, increase and decrease the synthesis of ROS (Chen et al., 2015b). Whether rabbit Nox5 behaves similarly is not yet known although the identified binding region for calmodulin in human Nox5 (aa 673–690, Nox5V2) is reasonably well-conserved in rabbit Nox5.

While the mechanisms regulating Nox5 activity have been well-characterized, comparatively little is known about the functional effects of Nox5 and in particular its role, if any, in physiology and pathophysiology. Increased expression of Nox5 in cancer and cardiovascular disease suggest an important role in pathophysiology, but in the absence of appropriate tools this hypothesis has not advanced beyond speculation. The first limitation is the lack of appropriate animal models that express Nox5. Rabbits have been used extensively as models of human cardiovascular diseases such as hypertension and atherosclerosis. The current study provides important resources that will facilitate investigation of a role of Nox5 in cardiovascular disease using rabbit models. Recent advances in genome editing such as CRISPR/Cas9 make rabbit Nox5 knockouts an important and achievable goal (Yan et al., 2014). Another limitation in identifying the significance of Nox5 is the lack of selective pharmacological tools. A number of compounds have been described that do inhibit Nox5 activity, however they also target other Nox isoforms and a remaining obstacle in the field is the development of specific Nox5 inhibitors (Chen et al., 2015b). To selectively disrupt rabbit Nox5 expression, we designed 4 miRNAs that specifically recognize rabbit Nox5 mRNA and identified one sequence, miRNA-1 that was most effective at reducing the expression of rabbit Nox5 and attendant superoxide production. The ability to effectively silence rabbit Nox5 should be advantageous in experiments using rabbit cells where Nox5 is suspected of contributing to altered cell signaling and function.

Construction of a phylogenetic tree provides additional information about the relationship between the rabbit and human Nox5. These results show that although rabbit Nox5 is highly similar to human Nox5, it is grouped into a distinct cluster. Surprisingly, a close genetic relationship was observed between the rabbit and armadillo, a placental mammal with leathery armor. Interestingly, the native American Nahuatl have long referred to Armadillos as “turtle-rabbits” (Karttunen, 1983) and well before any genetic relationship between rabbit and armadillo was appreciated. Phylogenetic analysis indicates that Nox5 originated from a eukaryotic ancestor during the Cambrian period of the Paleozoic era (He and Deem, 2010) and predates the other Nox isoforms. The reasons for why Nox5 was lost from rodent genomes, but not from other species, during the process of evolution remains elusive. In humans, Nox5 is expressed in testes, particularly in the pachytene spermatocytes where it is associated with maturing spermatids as well as in the uterus, ovaries, and placenta (Bánfi et al., 2001; Ritsick et al., 2007). Due to the lack of direct functional studies, it is not yet clear whether Nox5 has a meaningful role in reproduction. Several SNPs that encode non-functional variants of Nox5 are found at relatively high frequency in humans suggesting the possible existence of humans that lack a functional Nox5 (Wang et al., 2014). Whether fertility is affected by the loss of Nox5 function remains to be determined but a role for ROS has been well-demonstrated in sperm maturation, capacitation, regulation of intracellular pH, hyperactivation, and acrosomal exocytosis (de Lamirande and O'Flaherty, 2008). In addition, orthologs of Nox5 in Drosophila (dNox) are important for muscular contractions of the ovaries and egg laying (Ritsick et al., 2007). Although a general assumption might be that the loss of genetic material, or more specifically Nox5, implies a decrease in evolutionary fitness, the opposite hypothesis of “less is more” has been shown to be a potent driver of evolutionary change (Olson, 1999). The loss of Nox5 in a rodent ancestor may therefore have provided a survival or reproductive advantage. Coincidentally, a number of other genes have been lost from the genomes of rodents including CYP1D, and members of the ABC (ATP binding cassette) transporter (Dean and Annilo, 2005; Kawai et al., 2010).

In conclusion, we have determined the mRNA and protein sequence of rabbit Nox5 and characterized that the rabbit Nox5 gene is functional and regulated in a fashion similar to human Nox5 and also that it can be recognized by anti-human Nox5 polyclonal antibodies. Our study establishes the suitability of the rabbit as a model organism to further our understanding of the role of Nox5 in cardiovascular and other diseases.

## Author contributions

FC performed the Nox5 cloning and contributed to all of the experiments and wrote the original draft of the manuscript. CD contributed to the rationale of the paper and provided rabbit tissue. DF conceived experiments and wrote the manuscript, edited figures. CY performed the phylogenetic analysis.

## Funding

This work was supported by the National Institutes of Health RO1 HL124773 (DS, DF), P01 HL101902-01A1 (DF), 1R01HL125926-01A1 (DF) and NSFC 81570378 (FC). The funders had no role in study design, data collection and analysis, decision to publish, or preparation of the manuscript.

### Conflict of interest statement

The authors declare that the research was conducted in the absence of any commercial or financial relationships that could be construed as a potential conflict of interest.
