# Supplementary material for: Cloning, Characteristics, and Functional Analysis of Rabbit NADPH Oxidase 5
Source: Front Physiol. 2016 Jul 19;7:284. doi: 10.3389/fphys.2016.00284 (PMC4950256; doi:10.3389/fphys.2016.00284)
Supplement: Supplementary file 1 [file Image1.PDF]

```
                10        20        30        40        50        60        70        80
         ....|....|....|....|....|....|....|....|....|....|....|....|....|....|....|....|
rNOX5    ATGAGCTCACCGGGAGACCCTGCCCAACCAGGTCCCCAGGGCGGCAGAAGCACCCTGTCTGCCCAGGAGGACACCAAGTG
          M  S  S  P  G  D  P  A  Q  P  G  P  Q  G  G  R  S  T  L  S  A  Q  E  D  T  K  W
X1       ATGAGCTCACCGGGAGACCCTGCCCAACCAGGTCCCCAGGGCGGCAGAAGCACCCTGTCTGCCCAGGAGGACGCCAAGTG
          M  S  S  P  G  D  P  A  Q  P  G  P  Q  G  G  R  S  T  L  S  A  Q  E  D  A  K  W
X4       ATGAGCTCACCGGGAGACCCTGCCCAACCAGGTCCCCAGGGCGGCAGAAGCACCCTGTCTGCCCAGGAGGACGCCAAGTG
          M  S  S  P  G  D  P  A  Q  P  G  P  Q  G  G  R  S  T  L  S  A  Q  E  D  A  K  W
X2       ATGAGCTCACCGGGAGACCCTGCCCAACCAGGTCCCCAGGGCGGCAGAAGCACCCTGTCTGCCCAGGAGGACGCCAAGTG
          M  S  S  P  G  D  P  A  Q  P  G  P  Q  G  G  R  S  T  L  S  A  Q  E  D  A  K  W
X3       ATGAGCTCACCGGGAGACCCTGCCCAACCAGGTCCCCAGGGCGGCAGAAGCACCCTGTCTGCCCAGGAGGACGCCAAGTG
          M  S  S  P  G  D  P  A  Q  P  G  P  Q  G  G  R  S  T  L  S  A  Q  E  D  A  K  W

                90       100       110       120       130       140       150       160
         ....|....|....|....|....|....|....|....|....|....|....|....|....|....|....|....|
rNOX5    GCTGCGATGGGTGACGCACCAGTTTGAGGCCATTGCGGGAGAGGACCGGGAGATCAGCTTGCAGGAATTCAAGGCGGCTC
           L  R  W  V  T  H  Q  F  E  A  I  A  G  E  D  R  E  I  S  L  Q  E  F  K  A  A
X1       GCTGCGATGGGTGACACACCAGTTTGAGGCCATTGCGGGAGAGGACCGGGAGATCAGCTTGCAGGAATTCAAGGCGGCTC
           L  R  W  V  T  H  Q  F  E  A  I  A  G  E  D  R  E  I  S  L  Q  E  F  K  A  A
X4       GCTGCGATGGGTGACACACCAGTTTGAGGCCATTGCGGGAGAGGACCGGGAGATCAGCTTGCAGGAATTCAAGGCGGCTC
           L  R  W  V  T  H  Q  F  E  A  I  A  G  E  D  R  E  I  S  L  Q  E  F  K  A  A
X2       GCTGCGATGGGTGACACACCAGTTTGAGGCCATTGCGGGAGAGGACCGGGAGATCAGCTTGCAGGAATTCAAGGCGGCTC
           L  R  W  V  T  H  Q  F  E  A  I  A  G  E  D  R  E  I  S  L  Q  E  F  K  A  A
X3       GCTGCGATGGGTGACACACCAGTTTGAGGCCATTGCGGGAGAGGACCGGGAGATCAGCTTGCAGGAATTCAAGGCGGCTC
           L  R  W  V  T  H  Q  F  E  A  I  A  G  E  D  R  E  I  S  L  Q  E  F  K  A  A

               170       180       190       200       210       220       230       240
         ....|....|....|....|....|....|....|....|....|....|....|....|....|....|....|....|
rNOX5    TGAACGTGAAAGAGTCCTTCTTTGCGGAGCGATTCTTTGCCCTGTTCGACTCGGACAAGAGCGGCACCATCACCCTCCAG
         L  N  V  K  E  S  F  F  A  E  R  F  F  A  L  F  D  S  D  K  S  G  T  I  T  L  Q
X1       TGAACGTGAAAGAGTCCTTCTTTGCGGAGCGATTCTTTGCCCTGTTCGACTCGGACAAGAGCGGCACCATCACCCTCCAG
         L  N  V  K  E  S  F  F  A  E  R  F  F  A  L  F  D  S  D  K  S  G  T  I  T  L  Q
X4       TGAACGTGAAAGAGTCCTTCTTTGCGGAGCGATTCTTTGCCCTGTTCGACTCGGACAAGAGCGGCACCATCACCCTCCAG
         L  N  V  K  E  S  F  F  A  E  R  F  F  A  L  F  D  S  D  K  S  G  T  I  T  L  Q
X2       TGAACGTGAAAGAGTCCTTCTTTGCGGAGCGATTCTTTGCCCTGTTCGACTCGGACAAGAGCGGCACCATCACCCTCCAG
         L  N  V  K  E  S  F  F  A  E  R  F  F  A  L  F  D  S  D  K  S  G  T  I  T  L  Q
X3       TGAACGTGAAAGAGTCCTTCTTTGCGGAGCGATTCTTTGCCCTGTTCGACTCGGACAAGAGCGGCACCATCACCCTCCAG
         L  N  V  K  E  S  F  F  A  E  R  F  F  A  L  F  D  S  D  K  S  G  T  I  T  L  Q

               250       260       270       280       290       300       310       320
         ....|....|....|....|....|....|....|....|....|....|....|....|....|....|....|....|
rNOX5    AAGCTGCAGGAGGCGCTGACCCTGCTCATCCATGGGAGCCCTATGGACAAACTCAAGTTCCTTTTCCAGGTGTACGATGT
          K  L  Q  E  A  L  T  L  L  I  H  G  S  P  M  D  K  L  K  F  L  F  Q  V  Y  D  V
X1       GAGCTGCAGGAGGCGCTGACCCTGCTCATCCATGGGAGCCCTATGGACAAACTCAAGTTCCTTTTCCAGGTGTACGATGT
          E  L  Q  E  A  L  T  L  L  I  H  G  S  P  M  D  K  L  K  F  L  F  Q  V  Y  D  V
X4       GAGCTGCAGGAGGCGCTGACCCTGCTCATCCATGGGAGCCCTATGGACAAACTCAAGTTCCTTTTCCAGGTGTACGATGT
          E  L  Q  E  A  L  T  L  L  I  H  G  S  P  M  D  K  L  K  F  L  F  Q  V  Y  D  V
X2       GAGCTGCAGGAGGCGCTGACCCTGCTCATCCATGGGAGCCCTATGGACAAACTCAAGTTCCTTTTCCAGGTGTACGATGT
          E  L  Q  E  A  L  T  L  L  I  H  G  S  P  M  D  K  L  K  F  L  F  Q  V  Y  D  V
X3       GAGCTGCAGGAGGCGCTGACCCTGCTCATCCATGGGAGCCCTATGGACAAACTCAAGTTCCTTTTCCAGGTGTACGATGT
          E  L  Q  E  A  L  T  L  L  I  H  G  S  P  M  D  K  L  K  F  L  F  Q  V  Y  D  V

               330       340       350       360       370       380       390       400
         ....|....|....|....|....|....|....|....|....|....|....|....|....|....|....|....|
rNOX5    CGATGGC---------------------------------------------------------------AACGGCTCCA
           D  G                                                                  N  G  S
```

```
rNOX5  CGATGGC---------------------------------------------------------------AACGGCTCCA
         D  G                                                                  N  G  S
X1     CGATGGC---------------------------------------------------------------AACGGCTCCA
         D  G                                                                  N  G  S
X4     CGATGGCTTGCGGGCGCGGGCCGGCGAGGGCTCACGCGCGCCCGGCCTCCGCCCTGCGCCCTGGGTAGGCAACGGCTCCA
         D  G  L  R  A  R  A  G  E  G  S  R  A  P  G  L  R  P  A  P  W  V  G  N  G  S
X2     CGATGGC---------------------------------------------------------------AACGGCTCCA
         D  G                                                                  N  G  S
X3     CGATGGC---------------------------------------------------------------AACGGCTCCA
         D  G                                                                  N  G  S

                410       420       430       440       450       460       470       480
       ....|....|....|....|....|....|....|....|....|....|....|....|....|....|....|....|
rNOX5  TCGACGTGGACGAGCTGCGCACCGTGCTGCAGTCGTGCCTGCGCGAGAGCGCCCTCTCGCTGCCCGCCGAGAAGCTGGAC
       I  D  V  D  E  L  R  T  V  L  Q  S  C  L  R  E  S  A  L  S  L  P  A  E  K  L  D
X1     TCGACGTGGACGAGCTGCGCACCGTGCTGCAGTCGTGCCTGCGCGAGAGCGCCCTCTCGCTGCCCGCCGAGAAGCTGGAC
       I  D  V  D  E  L  R  T  V  L  Q  S  C  L  R  E  S  A  L  S  L  P  A  E  K  L  D
X4     TCGACGTGGACGAGCTGCGCACCGTGCTGCAGTCGTGCCTGCGCGAGAGCGCCCTCTCGCTGCCCGCCGAGAAGCTGGAC
       I  D  V  D  E  L  R  T  V  L  Q  S  C  L  R  E  S  A  L  S  L  P  A  E  K  L  D
X2     TCGACGTGGACGAGCTGCGCACCGTGCTGCAGTCGTGCCTGCGCGAGAGCGCCCTCTCGCTGCCCGCCGAGAAGCTGGAC
       I  D  V  D  E  L  R  T  V  L  Q  S  C  L  R  E  S  A  L  S  L  P  A  E  K  L  D
X3     TCGACGTGGACGAGCTGCGCACCGTGCTGCAGTCGTGCCTGCGCGAGAGCGCCCTCTCGCTGCCCGCCGAGAAGCTGGAC
       I  D  V  D  E  L  R  T  V  L  Q  S  C  L  R  E  S  A  L  S  L  P  A  E  K  L  D

                490       500       510       520       530       540       550       560
       ....|....|....|....|....|....|....|....|....|....|....|....|....|....|....|....|
rNOX5  CAGCTCACGCTGGCGCTCTTCCAGTCGGCCGACCGGGACGGCAGCGGGGCCATCACCTTCGACGAGCTCCGGGACGAGCT
        Q  L  T  L  A  L  F  Q  S  A  D  R  D  G  S  G  A  I  T  F  D  E  L  R  D  E  L
X1     CAGCTCACGCTGGCGCTCTTCCAGTCGGCCGACCAGGACGGCAGCGGGGCCATCACCTTCGACGAGCTCCGGGACGAGCT
        Q  L  T  L  A  L  F  Q  S  A  D  Q  D  G  S  G  A  I  T  F  D  E  L  R  D  E  L
X4     CAGCTCACGCTGGCGCTCTTCCAGTCGGCCGACCAGGACGGCAGCGGGGCCATCACCTTCGACGAGCTCCGGGACGAGCT
        Q  L  T  L  A  L  F  Q  S  A  D  Q  D  G  S  G  A  I  T  F  D  E  L  R  D  E  L
X2     CAGCTCACGCTGGCGCTCTTCCAGTCGGCCGACCAGGACGGCAGCGGGGCCATCACCTTCGACGAGCTCCGGGACGAGCT
        Q  L  T  L  A  L  F  Q  S  A  D  Q  D  G  S  G  A  I  T  F  D  E  L  R  D  E  L
X3     CAGCTCACGCTGGCGCTCTTCCAGTCGGCCGACCAGGACGGCAGCGGGGCCATCACCTTCGACGAGCTCCGGGACGAGCT
        Q  L  T  L  A  L  F  Q  S  A  D  Q  D  G  S  G  A  I  T  F  D  E  L  R  D  E  L

                570       580       590       600       610       620       630       640
       ....|....|....|....|....|....|....|....|....|....|....|....|....|....|....|....|
rNOX5  GCAGCGCTTCCCCGGGGTCCTGGAGAACCTCACCATCAGCGCCGCTCACTGGCTGACTCCGCCTGCCGCCCAGCCTCGCC
         Q  R  F  P  G  V  L  E  N  L  T  I  S  A  A  H  W  L  T  P  P  A  A  Q  P  R
X1     GCAGCGCTTCCCCGGGGTCCTGGAGAACCTCACCATCAGCGCCGCTCACTGGCTGACTCCGCCTGCCGCCCGGCCTCGCC
         Q  R  F  P  G  V  L  E  N  L  T  I  S  A  A  H  W  L  T  P  P  A  A  R  P  R
X4     GCAGCGCTTCCCCGGGGTCCTGGAGAACCTCACCATCAGCGCCGCTCACTGGCTGACTCCGCCTGCCGCCCGGCCTCGCC
         Q  R  F  P  G  V  L  E  N  L  T  I  S  A  A  H  W  L  T  P  P  A  A  R  P  R
X2     GCAGCGCTTCCCCGGGGTCCTGGAGAACCTCACCATCAGCGCCGCTCACTGGCTGACTCCGCCTGCCGCCCGGCCTCGCC
         Q  R  F  P  G  V  L  E  N  L  T  I  S  A  A  H  W  L  T  P  P  A  A  R  P  R
X3     GCAGCGCTTCCCCGGGGTCCTGGAGAACCTCACCATCAGCGCCGCTCACTGGCTGACTCCGCCTGCCGCCCGGCCTCGCC
         Q  R  F  P  G  V  L  E  N  L  T  I  S  A  A  H  W  L  T  P  P  A  A  R  P  R

                650       660       670       680       690       700       710       720
       ....|....|....|....|....|....|....|....|....|....|....|....|....|....|....|....|
rNOX5  CGCGCCGGCCACGCCTGCTGACCCCCGCCTACTGGCACAACCACCGCAGCCAGCTGCTCTGCCTGGCCGCCTACGCGGGC
       P  R  R  P  R  L  L  T  P  A  Y  W  H  N  H  R  S  Q  L  L  C  L  A  A  Y  A  G
X1     CGCGCCGGCCACGCCTGCTGACCCCCGCCTACTGGCACAACCACCGCAGCCAGCTGCTCTGCCTGGCCGCCTACGCGGGC
       P  R  R  P  R  L  L  T  P  A  Y  W  H  N  H  R  S  Q  L  L  C  L  A  A  Y  A  G
```

```
X4     CGCGCCGGCCACGCCTGCTGACCCCCGCCTACTGGCACAACCACCGCAGCCAGCTGCTCTGCCTGGCCGCCTACGCGGGC
       P  R  R  P  R  L  L  T  P  A  Y  W  H  N  H  R  S  Q  L  L  C  L  A  A  Y  A  G
X2     CGCGCCGGCCACGCCTGCTGACCCCCGCCTACTGGCACAACCACCGCAGCCAGCTGCTCTGCCTGGCCGCCTACGCGGGC
       P  R  R  P  R  L  L  T  P  A  Y  W  H  N  H  R  S  Q  L  L  C  L  A  A  Y  A  G
X3     CGCGCCGGCCACGCCTGCTGACCCCCGCCTACTGGCACAACCACCGCAGCCAGCTGCTCTGCCTGGCCGCCTACGCGGGC
       P  R  R  P  R  L  L  T  P  A  Y  W  H  N  H  R  S  Q  L  L  C  L  A  A  Y  A  G

                730       740       750       760       770       780       790       800
       ....|....|....|....|....|....|....|....|....|....|....|....|....|....|....|....|
rNOX5  CTCCACGTGTTGCTCTTCGCGCTGGCGGCCAGCGCGCACCGGACCCTGGGCGCCAGCGTCATGGTGGCCAAGGGCTGCGG
        L  H  V  L  L  F  A  L  A  A  S  A  H  R  T  L  G  A  S  V  M  V  A  K  G  C  G
X1     CTCCACGTGTTGCTCTTCGCGCTGGCGGCCAGCGCGCACCGGACCCTGGGCGCCAGCGTCATGGTGGCCAAGGGCTGCGG
        L  H  V  L  L  F  A  L  A  A  S  A  H  R  T  L  G  A  S  V  M  V  A  K  G  C  G
X4     CTCCACGTGTTGCTCTTCGCGCTGGCGGCCAGCGCGCACCGGACCCTGGGCGCCAGCGTCATGGTGGCCAAGGGCTGCGG
        L  H  V  L  L  F  A  L  A  A  S  A  H  R  T  L  G  A  S  V  M  V  A  K  G  C  G
X2     CTCCACGTGTTGCTCTTCGCGCTGGCGGCCAGCGCGCACCGGACCCTGGGCGCCAGCGTCATGGTGGCCAAGGGCTGCGG
        L  H  V  L  L  F  A  L  A  A  S  A  H  R  T  L  G  A  S  V  M  V  A  K  G  C  G
X3     CTCCACGTGTTGCTCTTCGCGCTGGCGGCCAGCGCGCACCGGACCCTGGGCGCCAGCGTCATGGTGGCCAAGGGCTGCGG
        L  H  V  L  L  F  A  L  A  A  S  A  H  R  T  L  G  A  S  V  M  V  A  K  G  C  G

                810       820       830       840       850       860       870       880
       ....|....|....|....|....|....|....|....|....|....|....|....|....|....|....|....|
rNOX5  CCAGTGCCTCAACTTCGACTGCAGCTTCATCGCGGTGTTCATGTTCAGGCGCTGCCTTACCTGGCTGCGGGCCACGTGGC
         Q  C  L  N  F  D  C  S  F  I  A  V  F  M  F  R  R  C  L  T  W  L  R  A  T  W
X1     CCAGTGCCTCAACTTCGACTGCAGCTTCATCGCGGTGCTCATGTTCAGGCGCTGCCTTACCTGGCTGCGGGCCACGTGGC
         Q  C  L  N  F  D  C  S  F  I  A  V  L  M  F  R  R  C  L  T  W  L  R  A  T  W
X4     CCAGTGCCTCAACTTCGACTGCAGCTTCATCGCGGTGCTCATGTTCAGGCGCTGCCTTACCTGGCTGCGGGCCACGTGGC
         Q  C  L  N  F  D  C  S  F  I  A  V  L  M  F  R  R  C  L  T  W  L  R  A  T  W
X2     CCAGTGCCTCAACTTCGACTGCAGCTTCATCGCGGTGCTCATGTTCAGGCGCTGCCTTACCTGGCTGCGGGCCACGTGGC
         Q  C  L  N  F  D  C  S  F  I  A  V  L  M  F  R  R  C  L  T  W  L  R  A  T  W
X3     CCAGTGCCTCAACTTCGACTGCAGCTTCATCGCGGTGCTCATGTTCAGGCGCTGCCTTACCTGGCTGCGGGCCACGTGGC
         Q  C  L  N  F  D  C  S  F  I  A  V  L  M  F  R  R  C  L  T  W  L  R  A  T  W

                890       900       910       920       930       940       950       960
       ....|....|....|....|....|....|....|....|....|....|....|....|....|....|....|....|
rNOX5  TGGCTCAGGTCCTGCCCCTGGACCGCAACATCCAGTTTCACCAGCTCATGGGCTACGTGGTGGTCGGGCTGTCCCTCGTG
       L  A  Q  V  L  P  L  D  R  N  I  Q  F  H  Q  L  M  G  Y  V  V  V  G  L  S  L  V
X1     TGGCTCAGGTCCTGCCCCTGGACCGCAACATCCAGTTTCACCAGCTCATGGGCTACGTGGTGGTCGGGCTGTCCCTCGTG
       L  A  Q  V  L  P  L  D  R  N  I  Q  F  H  Q  L  M  G  Y  V  V  V  G  L  S  L  V
X4     TGGCTCAGGTCCTGCCCCTGGACCGCAACATCCAGTTTCACCAGCTCATGGGCTACGTGGTGGTCGGGCTGTCCCTCGTG
       L  A  Q  V  L  P  L  D  R  N  I  Q  F  H  Q  L  M  G  Y  V  V  V  G  L  S  L  V
X2     TGGCTCAGGTCCTGCCCCTGGACCGCAACATCCAGTTTCACCAGCTCATGGGCTACGTGGTGGTCGGGCTGTCCCTCGTG
       L  A  Q  V  L  P  L  D  R  N  I  Q  F  H  Q  L  M  G  Y  V  V  V  G  L  S  L  V
X3     TGGCTCAGGTCCTGCCCCTGGACCGCAACATCCAGTTTCACCAGCTCATGGGCTACGTGGTGGTCGGGCTGTCCCTCGTG
       L  A  Q  V  L  P  L  D  R  N  I  Q  F  H  Q  L  M  G  Y  V  V  V  G  L  S  L  V

                970       980       990      1000      1010      1020      1030      1040
       ....|....|....|....|....|....|....|....|....|....|....|....|....|....|....|....|
rNOX5  CACACGGTGGCCCACGTGGTGAACTTTGCGCTGCAAGTTCACTCTGGAGCCAGCCCTTTCCAGTTCTGGGAGCTGCTGCT
        H  T  V  A  H  V  V  N  F  A  L  Q  V  H  S  G  A  S  P  F  Q  F  W  E  L  L  L
X1     CACACGGTGGCCCACGTGGTGAACTTTGCGCTGCAAGTTCACTCTGGAGCCAGCCCTTTCCAGTTCTGGGAGCTGCTGCT
        H  T  V  A  H  V  V  N  F  A  L  Q  V  H  S  G  A  S  P  F  Q  F  W  E  L  L  L
X4     CACACGGTGGCCCACGTGGTGAACTTTGCGCTGCAAGTTCACTCTGGAGCCAGCCCTTTCCAGTTCTGGGAGCTGCTGCT
        H  T  V  A  H  V  V  N  F  A  L  Q  V  H  S  G  A  S  P  F  Q  F  W  E  L  L  L
X2     CACACGGTGGCCCACGTGGTGAACTTTGCGCTGCAAGTTCACTCTGGAGCCAGCCCTTTCCAGTTCTGGGAGCTGCTGCT
        H  T  V  A  H  V  V  N  F  A  L  Q  V  H  S  G  A  S  P  F  Q  F  W  E  L  L  L
```

```
X3     CACACGGTGGCCCACGTGGTGAACTTTGCGCTGCAAGTTCACTCTGGAGCCAGCCCTTTCCAGTTCTGGGAGCTGCTGCT
        H  T  V  A  H  V  V  N  F  A  L  Q  V  H  S  G  A  S  P  F  Q  F  W  E  L  L  L

               1050      1060      1070      1080      1090      1100      1110      1120
       ....|....|....|....|....|....|....|....|....|....|....|....|....|....|....|....|
rNOX5  CACCACCAGGCCCGGCATCGGCTGGGTGCACGGCTTGGCCTCTCCGACAGGTGTCGCCCTGCTGCTGCTACTGCTGCTCA
         T  T  R  P  G  I  G  W  V  H  G  L  A  S  P  T  G  V  A  L  L  L  L  L  L  L
X1     CACCACCAGGCCCGGCATCGGCTGGGTGCACGGCTTGGCCTCTCCGACAGGTGTTGCCCTGCTGCTGCTACTGCTGCTCA
         T  T  R  P  G  I  G  W  V  H  G  L  A  S  P  T  G  V  A  L  L  L  L  L  L  L
X4     CACCACCAGGCCCGGCATCGGCTGGGTGCACGGCTTGGCCTCTCCGACAGGTGTTGCCCTGCTGCTGCTACTGCTGCTCA
         T  T  R  P  G  I  G  W  V  H  G  L  A  S  P  T  G  V  A  L  L  L  L  L  L  L
X2     CACCACCAGGCCCGGCATCGGCTGGGTGCACGGCTTGGCCTCTCCGACAGGTGTTGCCCTGCTGCTGCTACTGCTGCTCA
         T  T  R  P  G  I  G  W  V  H  G  L  A  S  P  T  G  V  A  L  L  L  L  L  L  L
X3     CACCACCAGGCCCGGCATCGGCTGGGTGCACGGCTTGGCCTCTCCGACAGGTGTTGCCCTGCTGCTGCTACTGCTGCTCA
         T  T  R  P  G  I  G  W  V  H  G  L  A  S  P  T  G  V  A  L  L  L  L  L  L  L

               1130      1140      1150      1160      1170      1180      1190      1200
       ....|....|....|....|....|....|....|....|....|....|....|....|....|....|....|....|
rNOX5  TGGTCGCCTGTGCCAGCACCTGCGTCCGCAGGAGCGGCCACTTTGAGGTGTTCTATTGGACCCACCTGGCCTACCTCCCC
       M  V  A  C  A  S  T  C  V  R  R  S  G  H  F  E  V  F  Y  W  T  H  L  A  Y  L  P
X1     TGGTCGCCTGTGCCAGCCCCTGCGTCCGCAGGAGCGGCCACTTTGAGGTGTTCTATTGGACCCACCTGGCCTACCTCCCC
       M  V  A  C  A  S  P  C  V  R  R  S  G  H  F  E  V  F  Y  W  T  H  L  A  Y  L  P
X4     TGGTCGCCTGTGCCAGCCCCTGCGTCCGCAGGAGCGGCCACTTTGAGGTGTTCTATTGGACCCACCTGGCCTACCTCCCC
       M  V  A  C  A  S  P  C  V  R  R  S  G  H  F  E  V  F  Y  W  T  H  L  A  Y  L  P
X2     TGGTCGCCTGTGCCAGCCCCTGCGTCCGCAGGAGCGGCCACTTTGAGGTGTTCTATTGGACCCACCTGGCCTACCTCCCC
       M  V  A  C  A  S  P  C  V  R  R  S  G  H  F  E  V  F  Y  W  T  H  L  A  Y  L  P
X3     TGGTCGCCTGTGCCAGCCCCTGCGTCCGCAGGAGCGGCCACTTTGAGGTGTTCTATTGGACCCACCTGGCCTACCTCCCC
       M  V  A  C  A  S  P  C  V  R  R  S  G  H  F  E  V  F  Y  W  T  H  L  A  Y  L  P

               1210      1220      1230      1240      1250      1260      1270      1280
       ....|....|....|....|....|....|....|....|....|....|....|....|....|....|....|....|
rNOX5  ATATGGCTTCTGCTCATCCTGCATGGACCCAATTTCTGGAAGTGGCTGCTGGTCCCTGGCACCCTGTTCTTCCTGGAGAA
        I  W  L  L  L  I  L  H  G  P  N  F  W  K  W  L  L  V  P  G  T  L  F  F  L  E  K
X1     ATATGGCTTCTGCTCATCCTGCATGGACCCAATTTCTGGAAGTGGCTGCTGGTCCCCGGCACCCTGTTCTTCCTGGAGAA
        I  W  L  L  L  I  L  H  G  P  N  F  W  K  W  L  L  V  P  G  T  L  F  F  L  E  K
X4     ATATGGCTTCTGCTCATCCTGCATGGACCCAATTTCTGGAAGTGGCTGCTGGTCCCCGGCACCCTGTTCTTCCTGGAGAA
        I  W  L  L  L  I  L  H  G  P  N  F  W  K  W  L  L  V  P  G  T  L  F  F  L  E  K
X2     ATATGGCTTCTGCTCATCCTGCATGGACCCAATTTCTGGAAGTGGCTGCTGGTCCCCGGCACCCTGTTCTTCCTGGAGAA
        I  W  L  L  L  I  L  H  G  P  N  F  W  K  W  L  L  V  P  G  T  L  F  F  L  E  K
X3     ATATGGCTTCTGCTCATCCTGCATGGACCCAATTTCTGGAAGTGGCTGCTGGTCCCCGGCACCCTGTTCTTCCTGGAGAA
        I  W  L  L  L  I  L  H  G  P  N  F  W  K  W  L  L  V  P  G  T  L  F  F  L  E  K

               1290      1300      1310      1320      1330      1340      1350      1360
       ....|....|....|....|....|....|....|....|....|....|....|....|....|....|....|....|
rNOX5  GGCTGTCGGACTGGCAGCGTCCCGCATGGAGGCCCTGTGCATCGTGGAAGTCAACCTCCTCCCCTCCAAGGTCACTCACC
         A  V  G  L  A  A  S  R  M  E  A  L  C  I  V  E  V  N  L  L  P  S  K  V  T  H
X1     GGCTGTCGGACTGGCAGCGTCCCGCATGGAGGCCCTGTGCATCGTGGAAGTCAACCTCCTCCCCTCCAAGGTCACTCACC
         A  V  G  L  A  A  S  R  M  E  A  L  C  I  V  E  V  N  L  L  P  S  K  V  T  H
X4     GGCTGTCGGACTGGCAGCGTCCCGCATGGAGGCCCTGTGCATCGTGGAAGTCAACCTCCTCCCCTCCAAGGTCACTCACC
         A  V  G  L  A  A  S  R  M  E  A  L  C  I  V  E  V  N  L  L  P  S  K  V  T  H
X2     GGCTGTCGGACTGGCAGCGTCCCGCATGGAGGCCCTGTGCATCGTGGAAGTCAACCTCCTCCCCTCCAAGGTCACTCACC
         A  V  G  L  A  A  S  R  M  E  A  L  C  I  V  E  V  N  L  L  P  S  K  V  T  H
X3     GGCTGTCGGACTGGCAGCGTCCCGCATGGAGGCCCTGTGCATCGTGGAAGTCAACCTCCTCCCCTCCAAGGTCACTCACC
         A  V  G  L  A  A  S  R  M  E  A  L  C  I  V  E  V  N  L  L  P  S  K  V  T  H

               1370      1380      1390      1400      1410      1420      1430      1440
```

```
                1370      1380      1390      1400      1410      1420      1430      1440
       ....|....|....|....|....|....|....|....|....|....|....|....|....|....|....|....|
rNOX5  TCCTCATCCGGCGGCCGCCTCTCTTCCACTACCGGCCAGGCGACTACTTGTACCTGAACGTCCCCACCATCGCGCGCTAC
       L  L  I  R  R  P  P  L  F  H  Y  R  P  G  D  Y  L  Y  L  N  V  P  T  I  A  R  Y
X1     TCCTCATCCGGCGGCCGCCTCTCTTCCACTACCGGCCAGGCGACTACTTGTACCTGAACGTCCCCACCATCGCGCGCTAC
       L  L  I  R  R  P  P  L  F  H  Y  R  P  G  D  Y  L  Y  L  N  V  P  T  I  A  R  Y
X4     TCCTCATCCGGCGGCCGCCTCTCTTCCACTACCGGCCAGGCGACTACTTGTACCTGAACGTCCCCACCATCGCGCGCTAC
       L  L  I  R  R  P  P  L  F  H  Y  R  P  G  D  Y  L  Y  L  N  V  P  T  I  A  R  Y
X2     TCCTCATCCGGCGGCCGCCTCTCTTCCACTACCGGCCAGGCGACTACTTGTACCTGAACGTCCCCACCATCGCGCGCTAC
       L  L  I  R  R  P  P  L  F  H  Y  R  P  G  D  Y  L  Y  L  N  V  P  T  I  A  R  Y
X3     TCCTCATCCGGCGGCCGCCTCTCTTCCACTACCGGCCAGGCGACTACTTGTACCTGAACGTCCCCACCATCGCGCGCTAC
       L  L  I  R  R  P  P  L  F  H  Y  R  P  G  D  Y  L  Y  L  N  V  P  T  I  A  R  Y

                1450      1460      1470      1480      1490      1500      1510      1520
       ....|....|....|....|....|....|....|....|....|....|....|....|....|....|....|....|
rNOX5  GAGTGGCACCCTTTCACCATCAGCAGTGCTCCCGAGCAGAAAGACACCATCTGGCTGCACGTCCGGTCGGAAGGCCAGTG
        E  W  H  P  F  T  I  S  S  A  P  E  Q  K  D  T  I  W  L  H  V  R  S  E  G  Q  W
X1     GAGTGGCACCCTTTCACCATCAGCAGTGCTCCCGAGCAGAAAGACACCATCTGGCTGCACGTCCGGTCGGAAGGCCAGTG
        E  W  H  P  F  T  I  S  S  A  P  E  Q  K  D  T  I  W  L  H  V  R  S  E  G  Q  W
X4     GAGTGGCACCCTTTCACCATCAGCAGTGCTCCCGAGCAGAAAGACACCATCTGGCTGCACGTCCGGTCGGAAGGCCAGTG
        E  W  H  P  F  T  I  S  S  A  P  E  Q  K  D  T  I  W  L  H  V  R  S  E  G  Q  W
X2     GAGTGGCACCCTTTCACCATCAGCAGTGCTCCCGAGCAGAAAGACACCATCTGGCTGCACGTCCGGTCGGAAGGCCAGTG
        E  W  H  P  F  T  I  S  S  A  P  E  Q  K  D  T  I  W  L  H  V  R  S  E  G  Q  W
X3     GAGTGGCACCCTTTCACCATCAGCAGTGCTCCCGAGCAGAAAGACACCATCTGGCTGCACGTCCGGTCGGAAGGCCAGTG
        E  W  H  P  F  T  I  S  S  A  P  E  Q  K  D  T  I  W  L  H  V  R  S  E  G  Q  W

                1530      1540      1550      1560      1570      1580      1590      1600
       ....|....|....|....|....|....|....|....|....|....|....|....|....|....|....|....|
rNOX5  GACAAACAGGCTGCACGAGTTCTTCCAGGCATCGGACCCTGTGGGCTTTGGTCCCAAGAGGCTATCGAGGAGTCTGAGGC
         T  N  R  L  H  E  F  F  Q  A  S  D  P  V  G  F  G  P  K  R  L  S  R  S  L  R
X1     GACAAACAGGCTGCACGAGTTCTTCCAGGCATCGGACCCTGTGGGCTTTGGTCCCAAGAGGCTATCGAGGAGTCTGAGGC
         T  N  R  L  H  E  F  F  Q  A  S  D  P  V  G  F  G  P  K  R  L  S  R  S  L  R
X4     GACAAACAGGCTGCACGAGTTCTTCCAGGCATCGGACCCTGTGGGCTTTGGTCCCAAGAGGCTATCGAGGAGTCTGAGGC
         T  N  R  L  H  E  F  F  Q  A  S  D  P  V  G  F  G  P  K  R  L  S  R  S  L  R
X2     GACAAACAGGCTGCACGAGTTCTTCCAGGCATCGGACCCTGTGGGCTTTGGTCCCAAGAGGCTATCGAGGAGTCTGAGGC
         T  N  R  L  H  E  F  F  Q  A  S  D  P  V  G  F  G  P  K  R  L  S  R  S  L  R
X3     GACAAACAGGCTGCACGAGTTCTTCCAGGCATCGGACCCTGTGGGCTTTGGTCCCAAGAGGCTATCGAGGAGTCTGAGGC
         T  N  R  L  H  E  F  F  Q  A  S  D  P  V  G  F  G  P  K  R  L  S  R  S  L  R

                1610      1620      1630      1640      1650      1660      1670      1680
       ....|....|....|....|....|....|....|....|....|....|....|....|....|....|....|....|
rNOX5  TGAGACAGAGCCAGAGGAGGCCCCAGGGTGTGCAGCCCCAGCCCGTCCATTCTGGAGCCCCAGCCCAGGGAACAGTGGCC
       L  R  Q  S  Q  R  R  P  Q  G  V  Q  P  Q  P  V  H  S  G  A  P  A  Q  G  T  V  A
X1     TGAGACAGAGCCAGAGGAGGCCCCAGGGTGTGCAGCCCCAGCCCGTCCATTCCGGGGCCCCAGCCCAGGGAACAGTGGCT
       L  R  Q  S  Q  R  R  P  Q  G  V  Q  P  Q  P  V  H  S  G  A  P  A  Q  G  T  V  A
X4     TGAGACAGAGCCAGAGGAGGCCCCAGGGTGTGCAGCCCCAGCCCGTCCATTCCGGGGCCCCAGCCCAGGGAACAGTGGCT
       L  R  Q  S  Q  R  R  P  Q  G  V  Q  P  Q  P  V  H  S  G  A  P  A  Q  G  T  V  A
X2     TGAGACAGAGCCAGAGGAGGCCCCAGGGTGTGCAGCCCCAGCCCGTCCATTCCGGGGCCCCAGCCCAGGGAACAGTGGCT
       L  R  Q  S  Q  R  R  P  Q  G  V  Q  P  Q  P  V  H  S  G  A  P  A  Q  G  T  V  A
X3     TGAGACAGAGCCAGAGGAGGCCCCAGGGTGTGCAGCCCCAGCCCGTCCATTCCGGGGCCCCAGCCCAGGGAACAGTGGCT
       L  R  Q  S  Q  R  R  P  Q  G  V  Q  P  Q  P  V  H  S  G  A  P  A  Q  G  T  V  A

                1690      1700      1710      1720      1730      1740      1750      1760
       ....|....|....|....|....|....|....|....|....|....|....|....|....|....|....|....|
rNOX5  GCCTCCAAGGACGCCATTGTGGACCTGGCGTCATACAGCGAAGGACCTGCCCAGCAGGGTGGTGAAGACTGGGCATGCAC
```

```
               1690      1700      1710      1720      1730      1740      1750      1760
       ....|....|....|....|....|....|....|....|....|....|....|....|....|....|....|....|
         A  S  K  D  A  I  V  D  L  A  S  Y  S  E  G  P  A  Q  Q  G  G  E  D  W  A  C  T
X1     GCCTCCAAGGACGCCATTGTGGACCTGGCGTCATACAGCGAAGGACCTGCCCAGCAGGGTGGTGAAGACTGGGCATGCAC
         A  S  K  D  A  I  V  D  L  A  S  Y  S  E  G  P  A  Q  Q  G  G  E  D  W  A  C  T
X4     GCCTCCAAGGACGCCATTGTGGACCTGGCGTCATACAGCGAAGGACCTGCCCAGCAGGGTGGTGAAGACTGGGCATGCAC
         A  S  K  D  A  I  V  D  L  A  S  Y  S  E  G  P  A  Q  Q  G  G  E  D  W  A  C  T
X2     GCCTCCAAGGACGCCATTGTGGACCTGGCGTCATACAGCGAAGGACCTGCCCAGCAGGGTGGTGAAGACTGGGCATGCAC
         A  S  K  D  A  I  V  D  L  A  S  Y  S  E  G  P  A  Q  Q  G  G  E  D  W  A  C  T
X3     GCCTCCAAGGACGCCATTGTGGACCTGGCGTCATACAGCGAAGGACCTGCCCAGCAGGGTGGTGAAGACTGGGCATGCAC
         A  S  K  D  A  I  V  D  L  A  S  Y  S  E  G  P  A  Q  Q  G  G  E  D  W  A  C  T

               1770      1780      1790      1800      1810      1820      1830      1840
       ....|....|....|....|....|....|....|....|....|....|....|....|....|....|....|....|
rNOX5  CAGGACTGAGAGAGAGGCTGCAGCACCAGGGACAGCCTCTTCCGCAGACCTGGCCCCCGAGATGCCCTCCGAGAGCCACC
          R  T  E  R  E  A  A  A  P  G  T  A  S  S  A  D  L  A  P  E  M  P  S  E  S  H
X1     CAGGACTGAGAGAGAGGCTGCAGCACCAGGGACAGCCTCTTCCGCAGACCTGGCCCCCGAGATGCCCTCCGAGAGCCACC
          R  T  E  R  E  A  A  A  P  G  T  A  S  S  A  D  L  A  P  E  M  P  S  E  S  H
X4     CAGGACTGAGAGAGAGGCTGCAGCACCAGGGACAGCCTCTTCCGCAGACCTGGCCCCCGAGATGCCCTCCGAGAGCCACC
          R  T  E  R  E  A  A  A  P  G  T  A  S  S  A  D  L  A  P  E  M  P  S  E  S  H
X2     CAGGACTGAGAGAGAGGCTGCAGCACCAGGGACAGCCTCTTCCGCAGACCTGGCCCCCGAGATGCCCTCCGAGAGCCACC
          R  T  E  R  E  A  A  A  P  G  T  A  S  S  A  D  L  A  P  E  M  P  S  E  S  H
X3     CAGGACTGAGAGAGAGGCTGCAGCACCAGGGACAGCCTCTTCCGCAGACCTGGCCCCCGAGATGCCCTCCGAGAGCCACC
          R  T  E  R  E  A  A  A  P  G  T  A  S  S  A  D  L  A  P  E  M  P  S  E  S  H

               1850      1860      1870      1880      1890      1900      1910      1920
       ....|....|....|....|....|....|....|....|....|....|....|....|....|....|....|....|
rNOX5  AGTTCTGCAACATTAAGTGCTACATCGACGGCCCTTATGGGAGCCCCACCCGCAGGATCTTCGCCGCAGAGCACGCGGTG
       Q  F  C  N  I  K  C  Y  I  D  G  P  Y  G  S  P  T  R  R  I  F  A  A  E  H  A  V
X1     AGTTCTGCAACATTAAGTGCTACATCGACGGCCCTTATGGGAGCCCCACCCGCAGGATCTTCGCCGCAGAGCACGCGGTG
       Q  F  C  N  I  K  C  Y  I  D  G  P  Y  G  S  P  T  R  R  I  F  A  A  E  H  A  V
X4     AGTTCTGCAACATTAAGTGCTACATCGACGGCCCTTATGGGAGCCCCACCCGCAGGATCTTCGCCGCAGAGCACGCGGTG
       Q  F  C  N  I  K  C  Y  I  D  G  P  Y  G  S  P  T  R  R  I  F  A  A  E  H  A  V
X2     AGTTCTGCAACATTAAGTGCTACATCGACGGCCCTTATGGGAGCCCCACCCGCAGGATCTTCGCCGCAGAGCACGCGGTG
       Q  F  C  N  I  K  C  Y  I  D  G  P  Y  G  S  P  T  R  R  I  F  A  A  E  H  A  V
X3     AGTTCTGCAACATTAAGTGCTACATCGACGGCCCTTATGGGAGCCCCACCCGCAGGATCTTCGCCGCAGAGCACGCGGTG
       Q  F  C  N  I  K  C  Y  I  D  G  P  Y  G  S  P  T  R  R  I  F  A  A  E  H  A  V

               1930      1940      1950      1960      1970      1980      1990      2000
       ....|....|....|....|....|....|....|....|....|....|....|....|....|....|....|....|
rNOX5  CTGATTGGGGCGGGCATTGGAATCACGCCCTTCGCCTCCATCCTGCAGAGCATCCTGTACAGGCACCAGCAGAGGAAGCG
         L  I  G  A  G  I  G  I  T  P  F  A  S  I  L  Q  S  I  L  Y  R  H  Q  Q  R  K  R
X1     CTGATTGGGGCGGGCATTGGAATCACGCCCTTCGCCTCCATCCTGCAGAGCATCCTGTACAGGCACCAGCAGAGGAAGCG
         L  I  G  A  G  I  G  I  T  P  F  A  S  I  L  Q  S  I  L  Y  R  H  Q  Q  R  K  R
X4     CTGATTGGGGCGGGCATTGGAATCACGCCCTTCGCCTCCATCCTGCAGAGCATCCTGTACAGGCACCAGCAGAGGAAGCG
         L  I  G  A  G  I  G  I  T  P  F  A  S  I  L  Q  S  I  L  Y  R  H  Q  Q  R  K  R
X2     CTGATTGGGGCGGGCATTGGAATCACGCCCTTCGCCTCCATCCTGCAGAGCATCCTGTACAGGCACCAGCAGAGGAAGCG
         L  I  G  A  G  I  G  I  T  P  F  A  S  I  L  Q  S  I  L  Y  R  H  Q  Q  R  K  R
X3     CTGATTGGGGCGGGCATTGGAATCACGCCCTTCGCCTCCATCCTGCAGAGCATCCTGTACAGGCACCAGCAGAGGAAGCG
         L  I  G  A  G  I  G  I  T  P  F  A  S  I  L  Q  S  I  L  Y  R  H  Q  Q  R  K  R

               2010      2020      2030      2040      2050      2060      2070      2080
       ....|....|....|....|....|....|....|....|....|....|....|....|....|....|....|....|
rNOX5  AGTTTGTCCCAAATGCCATCATTGCTGGTCTAGTGGCATCCAGGACGAGGACATGAAACTCCAAAAGGTGGACTTCATCT
          V  C  P  K  C  H  H  C  W  S  S  G  I  Q  D  E  D  M  K  L  Q  K  V  D  F  I
```

```
               2010      2020      2030      2040      2050      2060      2070      2080
          ....|....|....|....|....|....|....|....|....|....|....|....|....|....|....|....|
rNOX5     AGTTTGTCCCAAATGCCATCATTGCTGGTCTAGTGGCATCCAGGACGAGGACATGAAACTCCAAAAGGTGGACTTCATCT
            V  C  P  K  C  H  H  C  W  S  S  G  I  Q  D  E  D  M  K  L  Q  K  V  D  F  I
X1        AGTTTGTCCCAAATGCCAGCATTGCTGGACTAGTGGCATCCAGGACGAGGACATGAAACTTCAAAAGGTGGACTTCATCT
            V  C  P  K  C  Q  H  C  W  T  S  G  I  Q  D  E  D  M  K  L  Q  K  V  D  F  I
X4        AGTTTGTCCCAAATGCCAGCATTGCTGGACTAGTGGCATCCAGGACGAGGACATGAAACTTCAAAAGGTGGACTTCATCT
            V  C  P  K  C  Q  H  C  W  T  S  G  I  Q  D  E  D  M  K  L  Q  K  V  D  F  I
X2        AGTTTGTCCCAAATGCCAGCATTGCTGGACTAGTGGCATCCAGGACGAGGACATGAAACTTCAAAAGGTGGACTTCATCT
            V  C  P  K  C  Q  H  C  W  T  S  G  I  Q  D  E  D  M  K  L  Q  K  V  D  F  I
X3        AGTTTGTCCCAAATGCCAGCATTGCTGGACTAGTGGCATCCAGGACGAGGACATGAAACTTCAAAAGGTGGACTTCATCT
            V  C  P  K  C  Q  H  C  W  T  S  G  I  Q  D  E  D  M  K  L  Q  K  V  D  F  I

               2090      2100      2110      2120      2130      2140      2150      2160
          ....|....|....|....|....|....|....|....|....|....|....|....|....|....|....|....|
rNOX5     GGATCAACCGAGACCAGAGGGCTTTCGAGTGGTTTGTGAGCCTGCTGACCAGGCTGGAGATGGAGCAGGCCAAGGAAGCT
          W  I  N  R  D  Q  R  A  F  E  W  F  V  S  L  L  T  R  L  E  M  E  Q  A  K  E  A
X1        GGATCAACCGAGACCAGAGGGCTTTCGAGTGGTTTGTGAGCCTGCTGACCAGGCTGGAGATGGAGCAGGCCAAGGAAGCT
          W  I  N  R  D  Q  R  A  F  E  W  F  V  S  L  L  T  R  L  E  M  E  Q  A  K  E  A
X4        GGATCAACCGAGACCAGAGGGCTTTCGAGTGGTTTGTGAGCCTGCTGACCAGGCTGGAGATGGAGCAGGCCAAGGAAGCT
          W  I  N  R  D  Q  R  A  F  E  W  F  V  S  L  L  T  R  L  E  M  E  Q  A  K  E  A
X2        GGATCAACCGAGACCAGAGGGCTTTCGAGTGGTTTGTGAGCCTGCTGACCAGGCTGGAGATGGAGCAGGCCAAGGAAGCT
          W  I  N  R  D  Q  R  A  F  E  W  F  V  S  L  L  T  R  L  E  M  E  Q  A  K  E  A
X3        GGATCAACCGAGACCAGAGGGCTTTCGAGTGGTTTGTGAGCCTGCTGACCAGGCTGGAGATGGAGCAGGCCAAGGAAGCT
          W  I  N  R  D  Q  R  A  F  E  W  F  V  S  L  L  T  R  L  E  M  E  Q  A  K  E  A

               2170      2180      2190      2200      2210      2220      2230      2240
          ....|....|....|....|....|....|....|....|....|....|....|....|....|....|....|....|
rNOX5     GAGCAAGACCGATTCCTGGAGCTGCACATGTACATGACCTCTGCCCTGGGCAAGAACGACATGAAGGCCATTGGCCTGCA
           E  Q  D  R  F  L  E  L  H  M  Y  M  T  S  A  L  G  K  N  D  M  K  A  I  G  L  Q
X1        GAGCAAGACCGATTCCTGGAGCTGCACATGTACATGACCTCTGCCCTGGGCAAGAACGACATGAAGGCCATTGGCCTGCA
           E  Q  D  R  F  L  E  L  H  M  Y  M  T  S  A  L  G  K  N  D  M  K  A  I  G  L  Q
X4        GAGCAAGACCGATTCCTGGAGCTGCACATGTACATGACCTCTGCCCTGGGCAAGAACGACATGAAGGCCATTGGCCTGCA
           E  Q  D  R  F  L  E  L  H  M  Y  M  T  S  A  L  G  K  N  D  M  K  A  I  G  L  Q
X2        GAGCAAG--CAAATGAGAAGAATGTGAAATTCCACTGTTCTGCAAGTGATCAGGTGGGCCAAGAATGCTGTTCATGTCTG
           E  Q     A  N  E  K  N  V  K  F  H  C  S  A  S  D  Q  V  G  Q  E  C  C  S  C  L
X3        GAGCAAG--CAAATGAGAAGAATGTGAAATTCCACTGTTCTGCAAGTGATCAGTTTTACAGAAAGGACA--CAACGCAGG
           E  Q     A  N  E  K  N  V  K  F  H  C  S  A  S  D  Q  F  Y  R  K  D     T  T  Q

               2250      2260      2270      2280      2290      2300      2310      2320
          ....|....|....|....|....|....|....|....|....|....|....|....|....|....|....|....|
rNOX5     GATGGCCCTCGACCTCCTGGCCGAGAAGGAGAAGAAGGACTCCATCACAGGCCTCCAGACCCGCACACAGCCTGGCCGGC
            M  A  L  D  L  L  A  E  K  E  K  K  D  S  I  T  G  L  Q  T  R  T  Q  P  G  R
X1        GATGGCCCTCGACCTCCTGGCCGAGAAGGAGAAGAAGGACTCCATCACAGGCCTCCAGACCCGCACACAGCCTGGCCGGC
            M  A  L  D  L  L  A  E  K  E  K  K  D  S  I  T  G  L  Q  T  R  T  Q  P  G  R
X4        GATGGCCCTCGACCTCCTGGCCGAGAAGGAGAAGAAGGACTCCATCACAGGCCTCCAGACCCGCACACAGCCTGGCCGGC
            M  A  L  D  L  L  A  E  K  E  K  K  D  S  I  T  G  L  Q  T  R  T  Q  P  G  R
X2        ACTTTTATCTGTTCTGTCAGTTACCA--GAACAGGAGTGTTTAA------------------------------------
           T  F  I  C  S  V  S  Y  Q     N  R  S  V  *
X3        ACCCGTAG------------------------------------------------------------------------
          D  P  *

               2330      2340      2350      2360      2370      2380      2390      2400
          ....|....|....|....|....|....|....|....|....|....|....|....|....|....|....|....|
rNOX5     CGGACTGGAACAAGGTGTTCCAGAAAGTGGCCGCTGAGAAGAAGGGCAAGGTGCAGGTCTTCTTCTGCGGCTCCCCGGCC
```

```
       ....|....|....|....|....|....|....|....|....|....|....|....|....|....|....|....|
       P  D  W  N  K  V  F  Q  K  V  A  A  E  K  K  G  K  V  Q  V  F  F  C  G  S  P  A
X1     CGGACTGGAACAAGGTGTTCCAGAAAGTGGCCGCTGAGAAGAAGGGCAAGGTGCAGGTCTTCTTCTGTGGCTCCCCAGCC
       P  D  W  N  K  V  F  Q  K  V  A  A  E  K  K  G  K  V  Q  V  F  F  C  G  S  P  A
X4     CGGACTGGAACAAGGTGTTCCAGAAAGTGGCCGCTGAGAAGAAGGGCAAGGTGCAGGTCTTCTTCTGTGGCTCCCCAGCC
       P  D  W  N  K  V  F  Q  K  V  A  A  E  K  K  G  K  V  Q  V  F  F  C  G  S  P  A
X2     --------------------------------------------------------------------------------
X3     --------------------------------------------------------------------------------

                2410      2420      2430      2440      2450      2460
       ....|....|....|....|....|....|....|....|....|....|....|....|....|.
rNOX5  CTGGCCAAGGTGCTGCGGGCCCACTGCGCGGACTTCCGCTTCAGATTCTTCCAGGAGAACTTCTAG
        L  A  K  V  L  R  A  H  C  A  D  F  R  F  R  F  F  Q  E  N  F  *
X1     CTGGCCAAGGTGCTGCGGGCCCACTGCGCGGACTTCCGCTTCAGATTCTTCCAGGAGAACTTCTAG
        L  A  K  V  L  R  A  H  C  A  D  F  R  F  R  F  F  Q  E  N  F  *
X4     CTGGCCAAGGTGCTGCGGGCCCACTGCGCGGACTTCCGCTTCAGATTCTTCCAGGAGAACTTCTAG
        L  A  K  V  L  R  A  H  C  A  D  F  R  F  R  F  F  Q  E  N  F  *
X2     ------------------------------------------------------------------
X3     ------------------------------------------------------------------
```
