# Supplementary material for: Cloning, Characteristics, and Functional Analysis of Rabbit NADPH Oxidase 5
Source: Front Physiol. 2016 Jul 19;7:284. doi: 10.3389/fphys.2016.00284 (PMC4950256; doi:10.3389/fphys.2016.00284)
Supplement: Supplementary file 2 [file Image2.PDF]

```
               M---GDPAQ-GP-G-R-TMSAEEDARWLRW
                        10        20        30
hALPHA.PRO     MNTSGDPAQTGPEGCRGTMSAEEDARWLRW  30
hBETA.PRO      ------------------MSAEEDARWLRW  12
hDELTA.PRO     ------------------MSAEEDARWLRW  12
hEPSILON.PRO   ------------------------------  1
hGAMMA.PRO     MNTSGDPAQTGPEGCRGTMSAEEDARWLRW  30
RABBIT.pro     MSSPGDPAQPGPQGGRSTLSAQEDTKWLRW  30

               VTQQFKTIAGEDGEISLQEFKAALHVKESF
                        40        50        60
hALPHA.PRO     VTQQFKTIAGEDGEISLQEFKAALHVKESF  60
hBETA.PRO      VTQQFKTIAGEDGEISLQEFKAALHVKESF  42
hDELTA.PRO     VTQQFKTIAGEDGEISLQEFKAALHVKESF  42
hEPSILON.PRO   ------------------------------  1
hGAMMA.PRO     VTQQFKTIAGEDGEISLQEFKAALHVKESF  60
RABBIT.pro     VTHQFEAIAGEDREISLQEFKAALNVKESF  60

               FAERFFALFDSDRSGTITLQELQEALTLLI
                        70        80        90
hALPHA.PRO     FAERFFALFDSDRSGTITLQELQEALTLLI  90
hBETA.PRO      FAERFFALFDSDRSGTITLQELQEALTLLI  72
hDELTA.PRO     FAERFFALFDSDRSGTITLQELQEALTLLI  72
hEPSILON.PRO   ------------------------------  1
hGAMMA.PRO     FAERFFALFDSDRSGTITLQELQEALTLLI  90
RABBIT.pro     FAERFFALFDSDKSGTITLQKLQEALTLLI  90

               HGSPMDKLKFLFQVYDID------------
                       100       110       120
hALPHA.PRO     HGSPMDKLKFLFQVYDID------------  109
hBETA.PRO      HGSPMDKLKFLFQVYDID------------  91
hDELTA.PRO     HGSPMDKLKFLFQVYDIDVCARQGASAGTE  102
hEPSILON.PRO   ------------------------------  1
hGAMMA.PRO     HGSPMDKLKFLFQVYDIDVCARQGASAGTE  120
RABBIT.pro     HGSPMDKLKFLFQVYDVD------------  109
```

```
               ----------------GSGSIDPDELRTVL
                        130       140       150
hALPHA.PRO     ----------------GSGSIDPDELRTVL 122
hBETA.PRO      ----------------GSGSIDPDELRTVL 104
hDELTA.PRO     WGAGAGPHWASSPLGTGSGSIDPDELRTVL 132
hEPSILON.PRO   ------------------------------ 1
hGAMMA.PRO     WGAGAGPHWASSPLGTGSGSIDPDELRTVL 150
RABBIT.pro     ----------------GNGSIDVDELRTVL 122

               QSCLRESAISLPDEKLDQLTLALFESADAD
                        160       170       180
hALPHA.PRO     QSCLRESAISLPDEKLDQLTLALFESADAD 152
hBETA.PRO      QSCLRESAISLPDEKLDQLTLALFESADAD 134
hDELTA.PRO     QSCLRESAISLPDEKLDQLTLALFESADAD 162
hEPSILON.PRO   ------------------------------ 1
hGAMMA.PRO     QSCLRESAISLPDEKLDQLTLALFESADAD 180
RABBIT.pro     QSCLRESALSLPAEKLDQLTLALFQSADRD 152

               GNGAITFEELRDELQRFPGVMENLTISAAH
                        190       200       210
hALPHA.PRO     GNGAITFEELRDELQRFPGVMENLTISAAH 182
hBETA.PRO      GNGAITFEELRDELQRFPGVMENLTISAAH 164
hDELTA.PRO     GNGAITFEELRDELQRFPGVMENLTISAAH 192
hEPSILON.PRO   --------------------MENLTISAAH 10
hGAMMA.PRO     GNGAITFEELRDELQRFPGVMENLTISAAH 210
RABBIT.pro     GSGAITFDELRDELQRFPGVLENLTISAAH 182

               WLTAPAPRPRPRRPRQLTRAYWHNHRSQLF
                        220       230       240
hALPHA.PRO     WLTAPAPRPRPRRPRQLTRAYWHNHRSQLF 212
hBETA.PRO      WLTAPAPRPRPRRPRQLTRAYWHNHRSQLF 194
hDELTA.PRO     WLTAPAPRPRPRRPRQLTRAYWHNHRSQLF 222
hEPSILON.PRO   WLTAPAPRPRPRRPRQLTRAYWHNHRSQLF 40
hGAMMA.PRO     WLTAPAPRPRPRRPRQLTRAYWHNHRSQLF 240
RABBIT.pro     WLTPPAAQPRPRRPRLLTPAYWHNHRSQLL 212
```

```
               C L A T Y A G L H V L L F G L A A S A H R D L G A S V M V A
                                 250                 260                 270

hALPHA.PRO     C L A T Y A G L H V L L F G L A A S A H R D L G A S V M V A  242
hBETA.PRO      C L A T Y A G L H V L L F G L A A S A H R D L G A S V M V A  224
hDELTA.PRO     C L A T Y A G L H V L L F G L A A S A H R D L G A S V M V A  252
hEPSILON.PRO   C L A T Y A G L H V L L F G L A A S A H R D L G A S V M V A  70
hGAMMA.PRO     C L A T Y A G L H V L L F G L A A S A H R D L G A S V M V A  270
RABBIT.pro     C L A A Y A G L H V L L F A L A A S A H R T L G A S V M V A  242

               K G C G Q C L N F D C S F I A V L M L R R C L T W L R A T W
                                 280                 290                 300

hALPHA.PRO     K G C G Q C L N F D C S F I A V L M L R R C L T W L R A T W  272
hBETA.PRO      K G C G Q C L N F D C S F I A V L M L R R C L T W L R A T W  254
hDELTA.PRO     K G C G Q C L N F D C S F I A V L M L R R C L T W L R A T W  282
hEPSILON.PRO   K G C G Q C L N F D C S F I A V L M L R R C L T W L R A T W  100
hGAMMA.PRO     K G C G Q C L N F D C S F I A V L M L R R C L T W L R A T W  300
RABBIT.pro     K G C G Q C L N F D C S F I A V F M F R R C L T W L R A T W  272

               L A Q V L P L D Q N I Q F H Q L M G Y V V V G L S L V H T V
                                 310                 320                 330

hALPHA.PRO     L A Q V L P L D Q N I Q F H Q L M G Y V V V G L S L V H T V  302
hBETA.PRO      L A Q V L P L D Q N I Q F H Q L M G Y V V V G L S L V H T V  284
hDELTA.PRO     L A Q V L P L D Q N I Q F H Q L M G Y V V V G L S L V H T V  312
hEPSILON.PRO   L A Q V L P L D Q N I Q F H Q L M G Y V V V G L S L V H T V  130
hGAMMA.PRO     L A Q V L P L D Q N I Q F H Q L M G Y V V V G L S L V H T V  330
RABBIT.pro     L A Q V L P L D R N I Q F H Q L M G Y V V V G L S L V H T V  302

               A H T V N F V L Q A Q A E A S P F Q F W E L L L T T R P G I
                                 340                 350                 360

hALPHA.PRO     A H T V N F V L Q A Q A E A S P F Q F W E L L L T T R P G I  332
hBETA.PRO      A H T V N F V L Q A Q A E A S P F Q F W E L L L T T R P G I  314
hDELTA.PRO     A H T V N F V L Q A Q A E A S P F Q F W E L L L T T R P G I  342
hEPSILON.PRO   A H T V N F V L Q A Q A E A S P F Q F W E L L L T T R P G I  160
hGAMMA.PRO     A H T V N F V L Q A Q A E A S P F Q F W E L L L T T R P G I  360
RABBIT.pro     A H V V N F A L Q V H S G A S P F Q F W E L L L T T R P G I  332
```

```
                  GWVHGSASPTGVALLLLLLLMFICSSSCIR
                            370       380       390
hALPHA.PRO        GWVHGSASPTGVALLLLLLLMFICSSSCIR  362
hBETA.PRO         GWVHGSASPTGVALLLLLLLMFICSSSCIR  344
hDELTA.PRO        GWVHGSASPTGVALLLLLLLMFICSSSCIR  372
hEPSILON.PRO      GWVHGSASPTGVALLLLLLLMFICSSSCIR  190
hGAMMA.PRO        GWVHGSASPTGVALLLLLLLMFICSSSCIR  390
RABBIT.pro        GWVHGLASPTGVALLLLLLLMVACASTCVR  362
```

```
                  RSGHFEVFYWTHLSYLLVWLLLIFHGPNFW
                            400       410       420
hALPHA.PRO        RSGHFEVFYWTHLSYLLVWLLLIFHGPNFW  392
hBETA.PRO         RSGHFEVFYWTHLSYLLVWLLLIFHGPNFW  374
hDELTA.PRO        RSGHFEVFYWTHLSYLLVWLLLIFHGPNFW  402
hEPSILON.PRO      RSGHFEVFYWTHLSYLLVWLLLIFHGPNFW  220
hGAMMA.PRO        RSGHFEVFYWTHLSYLLVWLLLIFHGPNFW  420
RABBIT.pro        RSGHFEVFYWTHLAYLPIWLLLILHGPNFW  392
```

```
                  KWLLVPGILFFLEKAIGLAVSRMAAVCIME
                            430       440       450
hALPHA.PRO        KWLLVPGILFFLEKAIGLAVSRMAAVCIME  422
hBETA.PRO         KWLLVPGILFFLEKAIGLAVSRMAAVCIME  404
hDELTA.PRO        KWLLVPGILFFLEKAIGLAVSRMAAVCIME  432
hEPSILON.PRO      KWLLVPGILFFLEKAIGLAVSRMAAVCIME  250
hGAMMA.PRO        KWLLVPGILFFLEKAIGLAVSRMAAVCIME  450
RABBIT.pro        KWLLVPGTLFFLEKAVGLAASRMEALCIVE  422
```

```
                  VNLLPSKVTHLLIKRPPFFHYRPGDYLYLN
                            460       470       480
hALPHA.PRO        VNLLPSKVTHLLIKRPPFFHYRPGDYLYLN  452
hBETA.PRO         VNLLPSKVTHLLIKRPPFFHYRPGDYLYLN  434
hDELTA.PRO        VNLLPSKVTHLLIKRPPFFHYRPGDYLYLN  462
hEPSILON.PRO      VNLLPSKVTHLLIKRPPFFHYRPGDYLYLN  280
hGAMMA.PRO        VNLLPSKVTHLLIKRPPFFHYRPGDYLYLN  480
RABBIT.pro        VNLLPSKVTHLLIRRPPLFHYRPGDYLYLN  452
```

```
              IPTIARYEWHPFTISSAPEQKDTIWLHIRS
                       490       500       510
hALPHA.PRO    IPTIARYEWHPFTISSAPEQKDTIWLHIRS 482
hBETA.PRO     IPTIARYEWHPFTISSAPEQKDTIWLHIRS 464
hDELTA.PRO    IPTIARYEWHPFTISSAPEQKDTIWLHIRS 492
hEPSILON.PRO  IPTIARYEWHPFTISSAPEQKDTIWLHIRS 310
hGAMMA.PRO    IPTIARYEWHPFTISSAPEQKDTIWLHIRS 510
RABBIT.pro    VPTIARYEWHPFTISSAPEQKDTIWLHVRS 482

              QGQWTNRLYESFKASDPLGRGSKRLSRSVT
                       520       530       540
hALPHA.PRO    QGQWTNRLYESFKASDPLGRGSKRLSRSVT 512
hBETA.PRO     QGQWTNRLYESFKASDPLGRGSKRLSRSVT 494
hDELTA.PRO    QGQWTNRLYESFKASDPLGRGSKRLSRSVT 522
hEPSILON.PRO  QGQWTNRLYESFKASDPLGRGSKRLSRSVT 340
hGAMMA.PRO    QGQWTNRLYESFKASDPLGRGSKRLSRSVT 540
RABBIT.pro    EGQWTNRLHEFFQASDPVGFGPKRLSRSLR 512

              MRKSQRSSKGS-------------------
                       550       560       570
hALPHA.PRO    MRKSQRSSKGS------------------- 524
hBETA.PRO     MRKSQRSSKGS------------------- 506
hDELTA.PRO    MRKSQRSSKGS------------------- 534
hEPSILON.PRO  MRKSQRSSKGS------------------- 352
hGAMMA.PRO    MRKSQRSSKGS------------------- 552
RABBIT.pro    LRQSQRRPQGVQPQPVHSGAPAQGTVAASK 542

              ------------------------------
                       580       590       600
hALPHA.PRO    ------------------------------ 524
hBETA.PRO     ------------------------------ 506
hDELTA.PRO    ------------------------------ 534
hEPSILON.PRO  ------------------------------ 352
hGAMMA.PRO    ------------------------------ 552
RABBIT.pro    DAIVDLASYSEGPAQQGGEDWACTRTEREA 572
```

```
                 ------------EILLEKHHKFCNIKCYID
                          610       620       630
hALPHA.PRO       ------------EILLEKHHKFCNIKCYID  540
hBETA.PRO        ------------EILLEKHHKFCNIKCYID  522
hDELTA.PRO       ------------EILLEKHHKFCNIKCYID  550
hEPSILON.PRO     ------------EILLEKHHKFCNIKCYID  368
hGAMMA.PRO       ------------EILLEKHHKFCNIKCYID  568
RABBIT.pro       AAPGTASSADLAPEMPSESHQFCNIKCYID  602

                 GPYGTPTRRIFASEHAVLIGAGIGITPFAS
                          640       650       660
hALPHA.PRO       GPYGTPTRRIFASEHAVLIGAGIGITPFAS  570
hBETA.PRO        GPYGTPTRRIFASEHAVLIGAGIGITPFAS  552
hDELTA.PRO       GPYGTPTRRIFASEHAVLIGAGIGITPFAS  580
hEPSILON.PRO     GPYGTPTRRIFASEHAVLIGAGIGITPFAS  398
hGAMMA.PRO       GPYGTPTRRIFASEHAVLIGAGIGITPFAS  598
RABBIT.pro       GPYGSPTRRIFAAEHAVLIGAGIGITPFAS  632

                 ILQSIMYRHQKRKHTCPSCQHSWIEGVQDN
                          670       680       690
hALPHA.PRO       ILQSIMYRHQKRKHTCPSCQHSWIEGVQDN  600
hBETA.PRO        ILQSIMYRHQKRKHTCPSCQHSWIEGVQDN  582
hDELTA.PRO       ILQSIMYRHQKRKHTCPSCQHSWIEGVQDN  610
hEPSILON.PRO     ILQSIMYRHQKRKHTCPSCQHSWIEGVQDN  428
hGAMMA.PRO       ILQSIMYRHQKRKHTCPSCQHSWIEGVQDN  628
RABBIT.pro       ILQSILYRHQQRKRVCPKCHHCWSSGIQDE  662

                 -MKLHKVDFIWINRDQRSFEWFVSLLTKLE
                          700       710       720
hALPHA.PRO       -MKLHKVDFIWINRDQRSFEWFVSLLTKLE  629
hBETA.PRO        -MKLHKVDFIWINRDQRSFEWFVSLLTKLE  611
hDELTA.PRO       -MKLHKVDFIWINRDQRSFEWFVSLLTKLE  639
hEPSILON.PRO     -MKLHKVDFIWINRDQRSFEWFVSLLTKLE  457
hGAMMA.PRO       -MKLHKVDFIWINRDQRSFEWFVSLLTKLE  657
RABBIT.pro       DMKLQKVDFIWINRDQRAFEWFVSLLTRLE  692
```

```
               MDQAEEAQYGRFLELHMYMTSALGKNDMKA
                        730       740       750
hALPHA.PRO     MDQAEEAQYGRFLELHMYMTSALGKNDMKA 659
hBETA.PRO      MDQAEEAQYGRFLELHMYMTSALGKNDMKA 641
hDELTA.PRO     MDQAEEAQYGRFLELHMYMTSALGKNDMKA 669
hEPSILON.PRO   MDQAEEAQYGRFLELHMYMTSALGKNDMKA 487
hGAMMA.PRO     MDQAEEAQYGRFLELHMYMTSALGKNDMKA 687
RABBIT.pro     MEQAKEAEQDRFLELHMYMTSALGKNDMKA 722

               IGLQMALDLLANKEKKDSITGLQTRTQPGR
                        760       770       780
hALPHA.PRO     IGLQMALDLLANKEKKDSITGLQTRTQPGR 689
hBETA.PRO      IGLQMALDLLANKEKKDSITGLQTRTQPGR 671
hDELTA.PRO     IGLQMALDLLANKEKKDSITGLQTRTQPGR 699
hEPSILON.PRO   IGLQMALDLLANKEKKDSITGLQTRTQPGR 517
hGAMMA.PRO     IGLQMALDLLANKEKKDSITGLQTRTQPGR 717
RABBIT.pro     IGLQMALDLLAEKEKKDSITGLQTRTQPGR 752

               PDWSKVFQKVAAEKKGKVQVFFCGSPALAK
                        790       800       810
hALPHA.PRO     PDWSKVFQKVAAEKKGKVQVFFCGSPALAK 719
hBETA.PRO      PDWSKVFQKVAAEKKGKVQVFFCGSPALAK 701
hDELTA.PRO     PDWSKVFQKVAAEKKGKVQVFFCGSPALAK 729
hEPSILON.PRO   PDWSKVFQKVAAEKKGKVQVFFCGSPALAK 547
hGAMMA.PRO     PDWSKVFQKVAAEKKGKVQVFFCGSPALAK 747
RABBIT.pro     PDWNKVFQKVAAEKKGKVQVFFCGSPALAK 782

               VLKGHCEKFGFRFFQENF-
                        820
hALPHA.PRO     VLKGHCEKFGFRFFQENF             737
hBETA.PRO      VLKGHCEKFGFRFFQENF             719
hDELTA.PRO     VLKGHCEKFGFRFFQENF             747
hEPSILON.PRO   VLKGHCEKFGFRFFQENF             565
hGAMMA.PRO     VLKGHCEKFGFRFFQENF             765
RABBIT.pro     VLRAHCADFRFRFFQENF.            801
```
