# Supplementary material for: Cloning, Characteristics, and Functional Analysis of Rabbit NADPH Oxidase 5
Source: Front Physiol. 2016 Jul 19;7:284. doi: 10.3389/fphys.2016.00284 (PMC4950256; doi:10.3389/fphys.2016.00284)
Supplement: Supplementary file 3 [file Image3.PDF]

Accession ID	Nox5 [Species]
XM_013193173	Anser cygnoides domesticus
XM_012818807	Clupea harengus (Atlantic herring)
NM_001101137	Bos taurus (cattle)
NM_001305472	Gallus gallus (chicken)
XM_003952683	Pan troglodytes (chimpanzee)
XM_012664821	Propithecus coquereli (Coquerel's sifaka)
NM_001103218	Canis lupus familiaris (dog)
XM_004758597	Mustela putorius furo (domestic ferret)
XM_002932959	Xenopus (Silurana) tropicalis (western clawed frog)
XM_012768385	Microcebus murinus (gray mouse lemur)
NM_001291670	Monodelphis domestica (gray short-tailed opossum)
XM_001495665	Equus caballus (horse)
AF325189	Homo sapiens
XM_011968825	Mandrillus leucophaeus (drill)
XM_012867443	Fundulus heteroclitus (mummichog)
XM_012522406	Dasypus novemcinctus (nine-banded armadillo)
Current study	Rabbit Nox5
XM_001085148	Macaca mulatta (Rhesus monkey)
XM_012092619	Cercocebus atys (sooty mangabey)
XM_011607206	Takifugu rubripes (Fugu rubripes)
